# Antioxidant and Anticancer Activities of Synthesized Methylated and Acetylated Derivatives of Natural Bromophenols

**DOI:** 10.3390/antiox11040786

**Published:** 2022-04-15

**Authors:** Hui Dong, Li Wang, Meng Guo, Dimitrios Stagos, Antonis Giakountis, Varvara Trachana, Xiukun Lin, Yankai Liu, Ming Liu

**Affiliations:** 1 Key Laboratory of Marine Drugs, Ministry of Education, School of Medicine and Pharmacy, Ocean University of China, Qingdao 266003, China; 21190811109@stu.ouc.edu.cn (H.D.); 21190811062@stu.ouc.edu.cn (L.W.); 21200811191@stu.ouc.edu.cn (M.G.); 2 Laboratory for Marine Drugs and Bioproducts of Qingdao National Laboratory for Marine Science and Technology, Qingdao 266237, China; 3 Department of Biochemistry and Biotechnology, University of Thessaly, Biopolis, 41500 Larissa, Greece; stagkos@med.uth.gr (D.S.); agiakountis@uth.gr (A.G.); 4 Department of Biology, Faculty of Medicine, School of Health Sciences, University of Thessaly, Biopolis, 41500 Larissa, Greece; vtrachana@med.uth.gr; 5 Department of Pharmacology, School of Pharmacy, Southwest Medical University, 319 Zhongshan Road, Jiangyang, Luzhou 646000, China; xiukunlin@swmu.edu.cn

**Keywords:** bromophenol derivatives, antioxidant, anticancer, K562, HaCaT

## Abstract

Natural bromophenols are important secondary metabolites in marine algae. Derivatives of these bromophenol are potential candidates for the drug development due to their biological activities, such as antioxidant, anticancer, anti-diabetic and anti-inflammatory activity. In our present study, we have designed and synthesized a series of new methylated and acetylated bromophenol derivatives from easily available materials using simple operation procedures and evaluated their antioxidant and anticancer activities on the cellular level. The results showed that 2.,3-dibromo-1-(((2-bromo-4,5-dimethoxybenzyl)oxy)methyl)-4,5-dimethoxybenzene (**3b-9**) and (oxybis(methylene))bis(4-bromo-6-methoxy-3,1-phenylene) diacetate (**4b-3**) compounds ameliorated H_2_O_2_-induced oxidative damage and ROS generation in HaCaT keratinocytes. Compounds 2.,3-dibromo-1-(((2-bromo-4,5-dimethoxybenzyl)oxy)methyl)-4,5-dimethoxybenzene (**3b-9**) and (oxybis(methylene) )bis(4-bromo-6-methoxy-3,1-phenylene) diacetate (**4b-3**) also increased the TrxR1 and HO-1 expression while not affecting Nrf2 expression in HaCaT. In addition, compounds (oxybis(methylene)bis(2-bromo-6-methoxy-4,1-phenylene) diacetate (**4b-4**) inhibited the viability and induced apoptosis of leukemia K562 cells while not affecting the cell cycle distribution. The present work indicated that some of these bromophenol derivatives possess significant antioxidant and anticancer potential, which merits further investigation.

## 1. Introduction

Reactive oxygen species (ROS) are produced by physiological processes in living organisms and are necessary for normal cell functions, such as proliferation and differentiation [1]. In cancer cells, elevated ROS are needed for continuous and fast proliferation. For example, in human chronic myeloid leukemia K562 cells, ROS induce BCR-ABL signal transduction, and increase genetic instability that promotes cell proliferation [2]. However, in atopic dermatitis, premature skin aging and other diseases, the excessive production of cellular ROS may attenuate the antioxidant system and cause oxidative damage [3]. Therefore, for treating these oxidative stress-induced diseases, it is necessary to inhibit excessive ROS production by stimulating cellular antioxidant responses, such as the nuclear factor erythroid 2-related factor 2 (Nrf2) [4]. Normally, Nrf2 is suppressed by its negative regulator Kelch-like epichlorohydrin-associated protein 1 (Keap1) that directs it to the ubiquitin proteasome system for degradation [5]. Under oxidative stress, Nrf2 dissociates from Keap1, translocates to the nucleus and is subsequently activated and induces the expression of genes associated with cellular antioxidant defense. It is a feasible and promising strategy to activate Nrf2 by using small compounds in order to overcome oxidative damage. For example, in our previous studies, we have found that natural bromophenol compounds could modulate the activation of Nrf2 pathway [6], and therefore suggesting the possession of promising antioxidant activity. 

**Figure 1:**
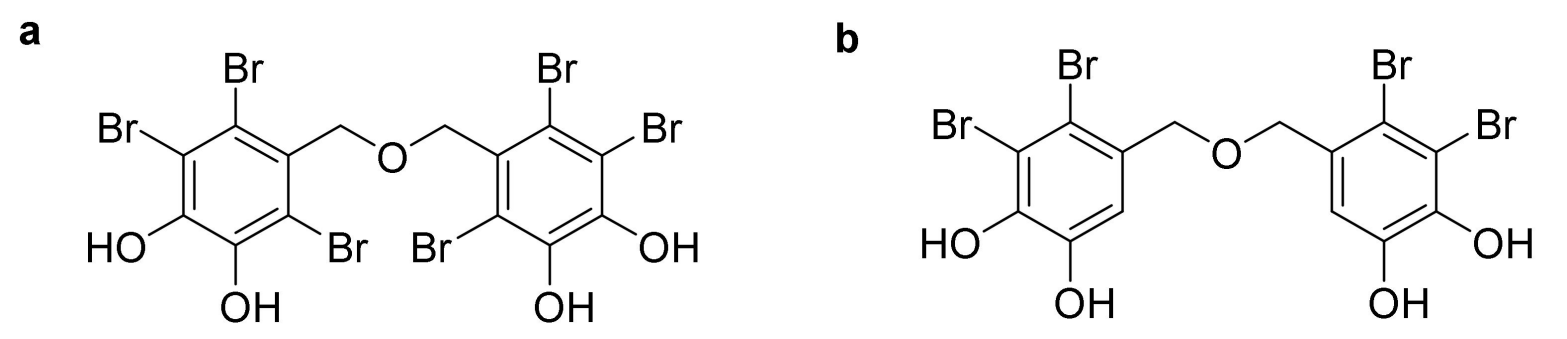
The chemical structure of natural bromophenol bis(2,3,6-tribromo-4,5-dihydroxybenzyl)ether (BTDE, **a**) and bis(2,3-dibromo-4,5-dihydroxybenzyl)ether (BDDE, **b**).

Natural bromophenols are important secondary metabolites in marine resources and have a variety of biological activities [7,8,9], especially anticancer and antioxidant, while some possess simultaneously both of them. For example, the bis (2,3,6-tribromo-4,5-dihydroxybenzyl) ether (BTDE, Figure 1a) is a typical representative molecule of natural bromophenols and is isolated from marine red alga *Symphyocladia latiuscula* [10]. BTDE showed both antioxidant activity and inhibition of cancer-related angiogenesis [11]. Another natural bromophenol bis (2,3-dibromo-4,5-dihydroxybenzyl) ether (BDDE, Figure 1b) also scavenges free radicals [12] and induces apoptosis of leukemia K562 cells [13]. It is believed that the OH groups in these molecules are important for the free radical scavenging activity, as shown in both DPPH and ABTS assays [14]. However, it is not clear how the modification of OH groups with the addition of methyl or ethyl groups affects bromophenols’ antioxidant and anticancer activities in cells.

In this study, we first synthesized 15 new derivatives of natural bromophenol compounds. Then, we assessed their cytoprotective effects against H_2_O_2_-induced damage in keratinocyte HaCaT cells. We also tested the effects of these compounds on the Nrf2 signaling pathway and the expression of the antioxidant proteins. Furthermore, considering the anticancer activity of natural bromophenol compounds, we also evaluated the cytotoxic activity of these derivatives against leukemia K562 cells and tried to find out the respective action mechanisms in the aspect of cell cycle arrest and apoptosis induction. 

## 2. Materials and Methods

### 2.1. Chemical Reagents, Purification, and Instrumentation

All the chemicals such as reagents and solvents were purchased from commercial sources and used as received without further purification, unless otherwise noted. Reactions were monitored by analytical thin layer chromatography (TLC), with visualization under UV light (254 nm). Column chromatography (CC) was performed with silica gel (300–400 mesh, Makall Group CO, LTD, Qingdao, Shandong, China). The ^1^H and ^13^C NMR spectra were recorded on a Bruker Avance NEO spectrometer (Bruker Co. Ltd, Billerica, Massachusetts, America). Chemical shifts are given in parts per million (ppm), and Tetramethyl silane (TMS) was used as an internal standard. Liquid chromatograph mass spectrometer (LC–MS) was performed using an Acquity UPLC H-Class coupled to a SQ Detector 2 mass spectrometer using a BEH C18 column (1.7 m, 2.1 50 mm, 1 mL/min) (Waters Corporation, Milford, MA, USA) and mobile phase for LC–MS was methanol and water.

### 2.2. Synthesis of 2,3-Dibromo-4-hydroxy-5-methoxybenzaldehyde (**1c**)

**Scheme 1:**
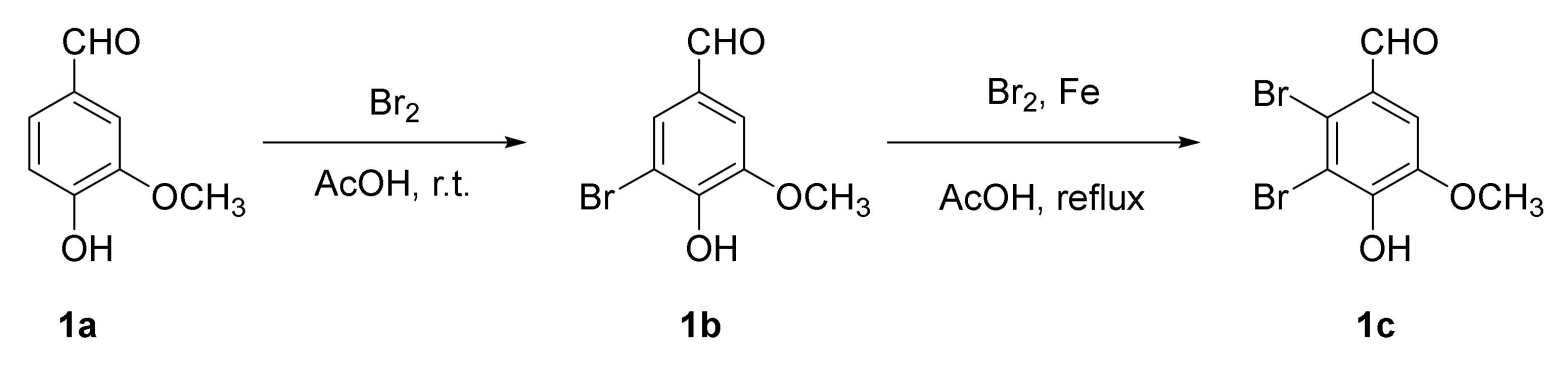
Synthesis of 2,3-dibromo-4-hydroxy-5-methoxybenzaldehyde (**1c**).

The synthesis of 2,3-dibromo-4-hydroxy-5-methoxybenzaldehyde (**1c**) consisted of two reactions (Scheme 1). Firstly, 3-bromo-4-hydroxy-5-methoxybenzaldehyde (**1b**) was synthesized. According to the literature [15], Vanillin (10 mmol, 1 eq.) was dissolved in acetic acid (20 mL), and then Br_2_ (11 mmol, 1.1eq.) was added dropwise to the solution at 25 °C. After stirring for 4 h, the white solid was collected by filtration and washed with cold water. The title compound (**1b**) (2.01 g, 8.7 mmol) was obtained in 87% yield after dried by oil pump vacuum, which was used for next step without further purification.

In the second reaction, synthesis of 2,3-dibromo-4-hydroxy-5-methoxybenzaldehyde (**1c**) compound was made according to literature [16]. This reaction included two steps. After the first reaction was complete, the iron filings (28 mg, 0.5 mmol) were directly added to the reaction solution of (**1b**) in acetic acid, and then Br_2_ (1.232 mL, 24 mmol) was added by drops. After being heated at reflux overnight, TLC analysis (Hex/EtOAc = 3:1) showed the complete consumption of compound (**1c**). The gray solid was collected by filtration and washed with cold water. After dried by oil pump vacuum, the title compound (**1c**) (3.42 g, 7.4 mmol) was obtained in 74% yield (over two steps), which can be directly used for the next step. 

### 2.3. Synthesis of 2-Bromo-3-hydroxy-4-methoxybenzaldehyde (**2b**)

**Scheme 2:**
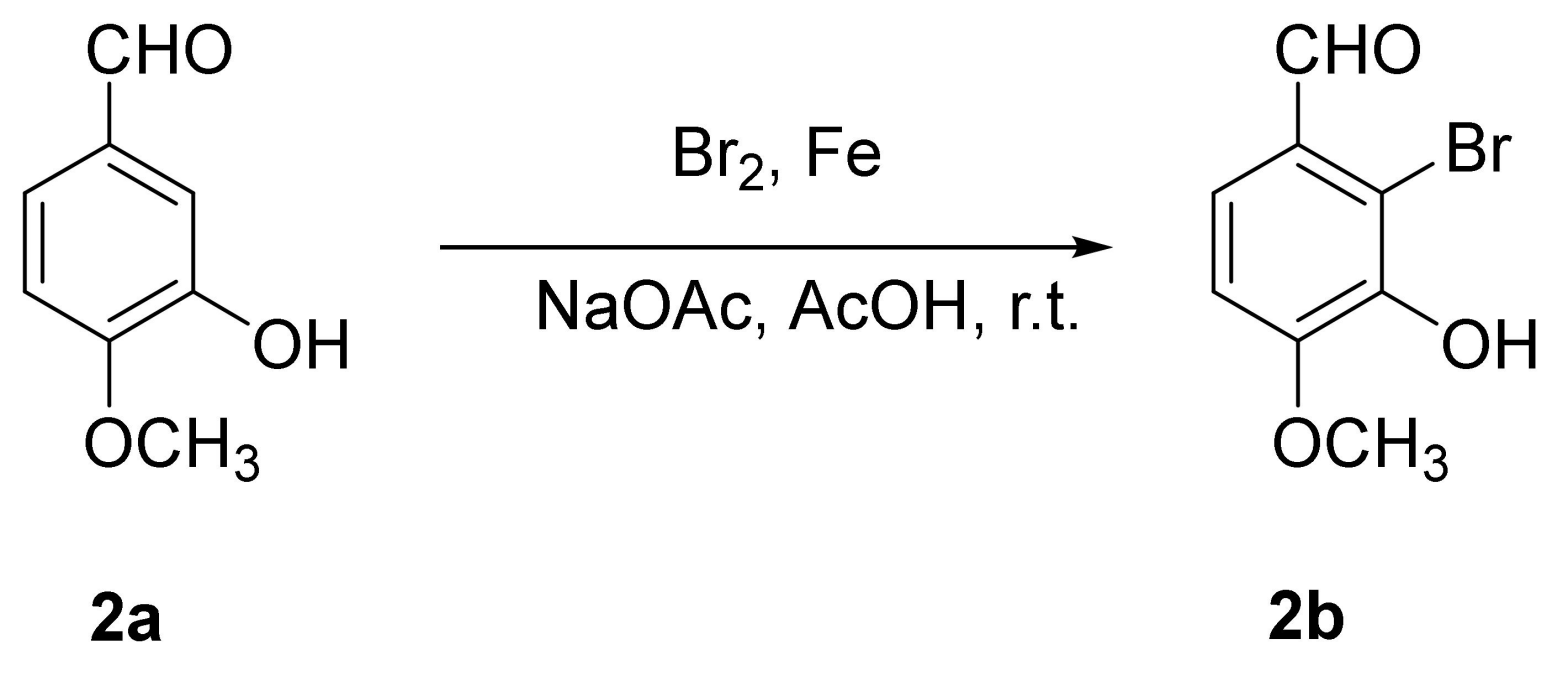
Synthesis of 2-bromo-3-hydroxy-4-methoxybenzaldehyde (**2b**). r.t., room temperature.

Synthesis of 2-bromo-3-hydroxy-4-methoxybenzaldehyde (**2b**) was based on a slightly modified literature procedure (Scheme 2) [17]. The isovanillin (10 mmol, 1eq.), anhydrous NaOAc (20 mmol, 2eq.) and iron filings (1 mmol, 0.1eq.) were successively added to acetic acid (20 mL). Next, Br_2_ (11 mmol, 1.1eq.) was slowly added dropwise into the above mixture at room temperature (Scheme 2). After complete consumption of (**2a**), ice-water (100 mL) was added to the reaction mixture, stirred for more 30 min. The solid product was obtained by filtration and recrystallized from EtOH to give **2b** (1.92 g, 83%) as a gray solid.

### 2.4. General Procedure of Methyl Bromophenol Derivatives

**Scheme 3:**
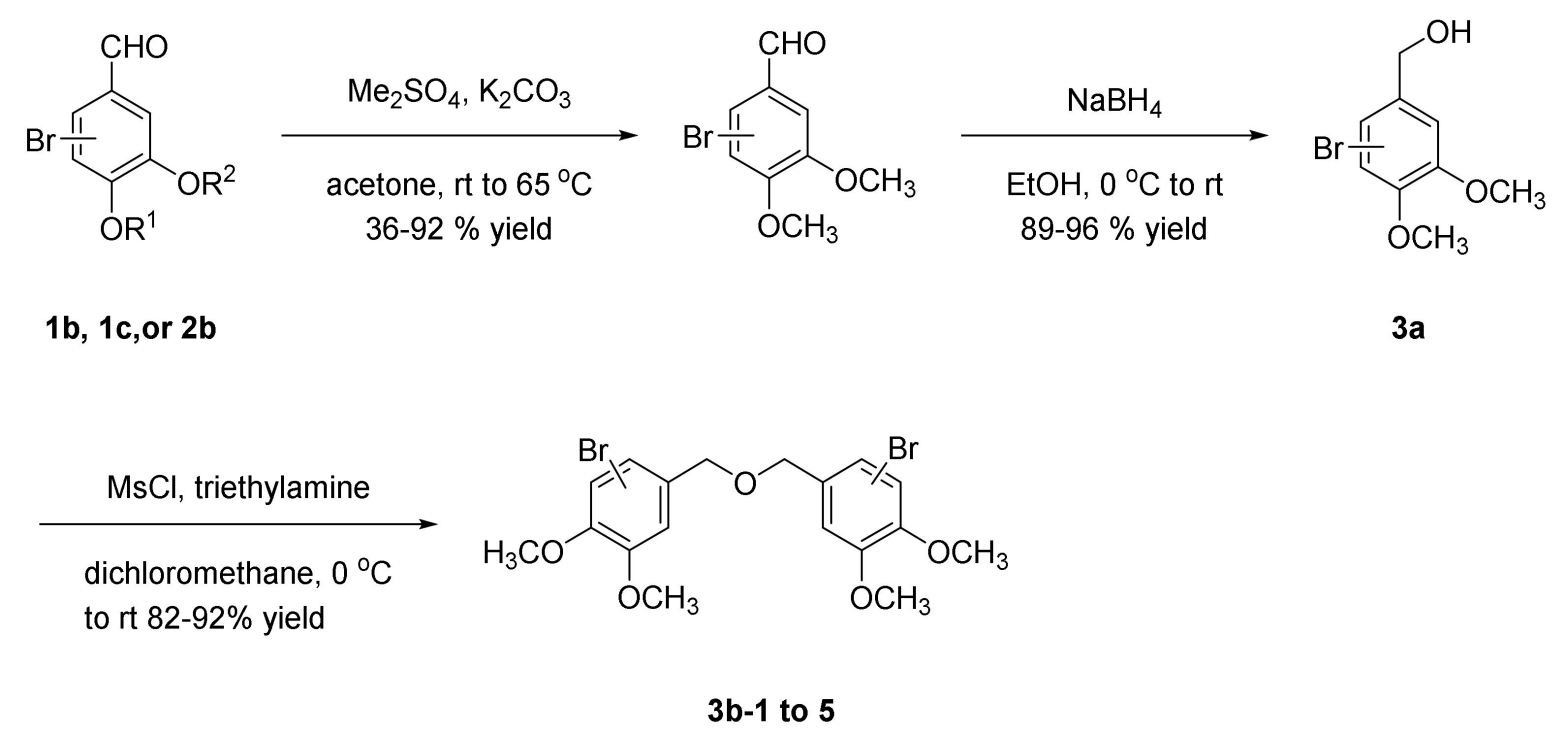
General procedure of methyl bromophenol derivatives.

To a 5 mL round-bottom flask, anhydrous K_2_CO_3_ (2 mmol, 2 eq.) was added, followed by a solution of one of the compounds (**1b**, **1c**, **2b**) and another commercial compound (1 mmol, 1 eq.) in acetone (2 mL). The reaction solution was stirred at room temperature. To this stirring mixture, Me_2_SO_4_ (2 mmol, 2 eq.) was added. The reaction was left to stir vigorously at 45 °C for 6 h (Scheme 3). The mixture was filtered through a Celite pad and the filtrate was concentrated to give the crude product. The crude product was chromatographed on silica gel (Hex/EtOAc) to give the compound of methylation (36–96% yield).

To a solution of methylation compound (1 mmol, 1 eq.) in EtOH (2 mL), NaBH_4_ (1.5 mmol, 1.5 eq.) was added at 0 °C (Scheme 3). After the substrates were completely consumed (checked by TLC monitoring), saturated NH_4_Cl solution was added to the reaction mixture, and the obtained residue was extracted with EtOAc (20 mL × 3). The combined organic layers were dried over Na_2_SO_4_ and concentrated in vacuo. The residue was purified by silica-gel column chromatography (Hex/EtOAc) to afford corresponding reduced compounds.

To a solution of reduced compounds (1 mmol, 1 eq.) and Et_3_N (1.2 mmol, 1.2 eq.) in dry CH_2_Cl_2_, MsCl (1.1 mmol, 1.1 eq.) was added dropwise at 0 °C. The reaction mixture was warmed to room temperature and stirred (Scheme 3). After the substrate was completely consumed (checked by TLC monitoring), the mixture was poured into cool water and the extracted with EtOAc (20 mL × 3). The combined organic layers were dried over Na_2_SO_4_ and concentrated in vacuo. The crude product was chromatographed on silica gel (Hex/EtOAc) to give the compound.

#### 2.4.1. Bis(3,4-dimethoxybenzyl)ether (**3b-1**)


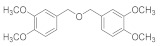


85% yield of white solid. **^1^**H NMR (400 MHz, Chloroform-d) δ 6.91 (d, *J =* 1.8 Hz, 2H), 6.89 (dd, *J =* 8.1, 1.9 Hz, 2H), 6.84 (d, *J =* 8.1 Hz, 2H), 4.47 (s, 4H), 3.88 (d, *J =* 1.7 Hz, 12H). ^13^C NMR (101 MHz, Chloroform-d) δ 149.07, 148.65, 130.86, 120.49, 111.23, 110.92, 71.80, 55.95.

#### 2.4.2. Bis(2,3-dibromo-4,5-dimethoxybenzene)ether (**3b-2**)


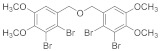


82% yield white solid. ^1^H NMR (400 MHz, Chloroform-d) δ 7.12 (s, 2H), 4.78 – 4.58 (m, 4H), 3.88 (s, 6H), 3.85 (s, 6H). ^13^C NMR (101 MHz, Chloroform-d) δ 152.65, 147.02, 134.80, 121.90, 115.49, 111.90, 73.34, 60.58, 56.24.

#### 2.4.3. Bis(2-Bromo-4,5-dimethoxybenzene)ether (**3b-3**)


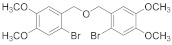


92% yield of white solid. ^1^H NMR (400 MHz, Chloroform-d) δ 7.04 (s, 2H), 7.02 (s, 2H), 4.62 (s, 4H), 3.87 (d, *J =* 1.1 Hz, 12H). ^13^C NMR (101 MHz, Chloroform-d) δ 149.82, 148.65, 128.61, 115.59, 114.55, 113.26, 56.26, 56.15, 46.52.

#### 2.4.4. Bis(3-Bromo-4,5-dimethoxybenzene)ether (**3b-4**)


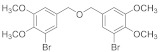


86% yield of white solid. ^1^H NMR (400 MHz, Chloroform-d) δ 7.12 (d, *J =* 1.9 Hz, 2H), 6.86 (d, *J =* 1.9 Hz, 2H), 4.45 (s, 4H), 3.86 (d, *J =* 9.1 Hz, 12H). ^13^C NMR (101 MHz, Chloroform-*d*) δ 153.79, 145.99, 135.16, 123.97, 117.54, 111.14, 71.47, 60.59, 56.11.

#### 2.4.5. Bis(2,3,6-Tribromo-4,5-dihydroxybenzyl)ether (**3b-5**)


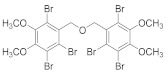


85% yield of white solid. ^1^H NMR (400 MHz, Chloroform-d) δ 5.03 (s, 4H), 3.87 (d, *J =* 2.3 Hz, 12H). ^13^C NMR (101 MHz, Chloroform-d) δ 152.27, 150.73, 133.63, 45,124.32, 122.15, 121.87, 73.89, 60.90, 60.83.

**Scheme 4:**
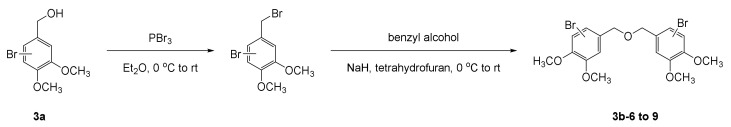
General procedure of asymmetric methyl bromophenol derivatives.

PBr_3_ (1.1 mmol, 1.1 eq.) at 0 °C under an N_2_ atmosphere was added to a stirred solution of alcohol (1.0 mmol, 1 eq.) in 2 mL of Et_2_O (Scheme 4). The solution was stirred at the rt (TLC monitoring). Thereafter, the solvent was evaporated to obtain a residue, which was partitioned between ethyl acetate (30 mL) and water (30 mL). The organic layer was separated, dried (Na_2_SO_4_) and evaporated. The crude product was chromatographed on silica gel (Hex/EtOAc) to give the compound.

To a solution of the corresponding alcohol (1 mmol, 1 eq.) in tetrahydrofuran(THF, 2 mL), was added to NaH (1.5 mmol, 60% in paraffin oil) at 0 °C under argon. After the reaction mixture was stirred for 30 min, benzyl bromide (1.2 mmol, 1.2 eq.) was added to the reaction mixture at 0 °C and the solution was stirred at room temperature (Scheme 4). Then, the reaction mixture was quenched with H_2_O (10 mL) and extracted with ethyl acetate (20 mL × 2). The combined organic layers were dried over Na_2_SO_4_ and concentrated under vacuum. The residue was purified by silica-gel column chromatography (Hex/EtOAc) to give the corresponding benzyl ether derivatives.

#### 2.4.6. 2,3-Dibromo-1-(((3-bromo-4,5-dimethoxybenzyl)oxy)methyl)-4,5-dimethoxybenzene (**3b-6**)


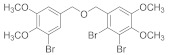


89% yield of white solid. ^1^H NMR (400 MHz, Chloroform-d) δ 7.15 (d, *J =* 1.8 Hz, 1H), 7.09 (s, 1H), 6.89 (d, *J =* 1.9 Hz, 1H), 4.60 (s, 2H), 4.55 (s, 2H), 3.88 (d, *J =* 3.6 Hz, 6H), 3.85 (d, *J =* 1.9 Hz, 6H). ^13^C NMR (101 MHz, Chloroform-*d*) δ 153.79, 152.68, 147.00, 146.07, 135.03, 134.95, 123.91, 121.89, 117.59, 115.55, 111.90, 111.11, 72.81, 72.09, 60.61, 60.57, 56.24, 56.12.

#### 2.4.7. 1-Bromo-5-(((2-bromo-4,5-dimethoxybenzyl)oxy)methyl)-2,3-dimethoxybenzene (**3b-7**)


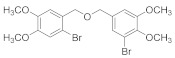


88% yield of white solid. ^1^H NMR (400 MHz, Chloroform-d) δ 7.14 (d, *J =* 1.8 Hz, 1H), 7.02 (s, 1H), 6.99 (s, 1H), 6.90 (d, *J =* 1.9 Hz, 1H), 4.56 (s, 2H), 4.51 (s, 2H), 3.89 – 3.86 (m, 9H), 3.84 (s, 3H). ^13^C NMR (101 MHz, Chloroform-d) δ 153.75, 149.13, 148.54, 145.97, 135.33, 129.24, 123.96, 117.52, 115.42, 113.39, 112.43, 111.17, 71.65, 71.59, 60.60, 56.24, 56.10.

#### 2.4.8. 1-Bromo-5-(((3,4-dimethoxybenzyl)oxy)methyl)-2,3-dimethoxybenzene (**3b-8**)


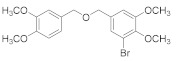


78% yield of white solid. ^1^H NMR (400 MHz, Chloroform-d) δ 7.12 (d, *J =* 1.8 Hz, 1H), 6.91 (d, *J =* 1.8 Hz, 1H), 6.89 – 6.82 (m, 3H), 4.46 (d, *J =* 21.2 Hz, 4H), 3.89 (d, *J =* 3.2 Hz, 6H), 3.85 (d, *J =* 7.3 Hz, 6H). ^13^C NMR (101 MHz, Chloroform-d) δ 153.74, 149.10, 148.76, 145.86, 135.56, 130.45, 123.93, 120.55, 117.48, 111.23, 111.13, 110.97, 72.23, 70.98, 60.57, 56.08, 55.95, 55.87.

#### 2.4.9. 2.,3-Dibromo-1-(((2-bromo-4,5-dimethoxybenzyl)oxy)methyl)-4,5-dimethoxybenzene (**3b-9**)


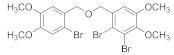


79% yield of Colorless oil. ^1^H NMR (400 MHz, Chloroform-d) δ 7.14 (s, 1H), 7.02 (d, *J =* 1.5 Hz, 2H), 4.65 (s, 4H), 3.87 (d, *J =* 1.1 Hz, 10H), 3.84 (s, 2H). ^13^C NMR (101 MHz, Chloroform-d) δ 152.65, 149.18, 148.52, 146.90, 135.13, 129.11, 121.81, 115.43, 115.36, 113.33, 112.44, 111.84, 72.86, 72.15, 60.56, 56.24, 56.21, 56.10.

### 2.5. General Procedure of Acetylation of Bromophenol Derivatives

One of the compounds (**1b**, **1c**, **2b** or a commercial compound, 1 mmol) and catalyst dimethylaminopyridine were added to the pyridine (2 mL). Acetic anhydride (0.104 mL, 1.1 mmol) was added by dropwise into the above mixture at 0 °C. After stirring the reaction mixture until the substrate was completely consumed (checked by TLC monitoring), the reaction mixture was warmed to room temperature. The slurry was poured into 30 mL 3M HCl and diluted with 20 mL EtOAc. The layers separated and the aqueous layer was extracted with 30 mL × 3 EtOAc, and the combined organics were washed with brine, dried over Na_2_SO_4_, filtered, and concentrated in vacuo. Then, the crude product was chromatographed on silica gel (Hex/EtOAc) to give the compound **4b**.

#### 2.5.1. (Oxybis(methylene))bis(2-bromo-6-methoxy-3,1-phenylene) diacetate (**4b-1**)


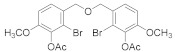


62% yield of white solid. ^1^H NMR (400 MHz, Chloroform-d) δ 7.33 (d, *J =* 8.5 Hz, 1H), 6.91 (d, *J =* 8.5 Hz, 1H), 4.69 (s, 2H), 3.83 (s, 3H), 2.37 (s, 3H). ^13^C NMR (101 MHz, Chloroform-d) δ 167.87, 152.51, 138.31, 129.56, 128.37, 119.56, 111.06, 56.31, 46.20, 20.48.

#### 2.5.2. (Oxybis(methylene))bis(2,3-dibromo-6-methoxy-4,1-phenylene)diacetate (**4b-2**)


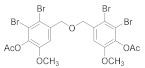


76% yield of white solid. ^1^H NMR (400 MHz, Chloroform-d) δ 7.11 (s, 2H), 4.73 (s, 4H), 3.86 (s, 6H), 2.37 (s, 6H). ^13^C NMR (101 MHz, Chloroform-d) δ 167.56, 151.53, 138.99, 136.23, 121.95, 117.18, 113.03, 56.45, 47.47, 20.48.

#### 2.5.3. (Oxybis(methylene))bis(4-bromo-6-methoxy-3,1-phenylene) diacetate (**4b-3**)


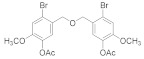


63% yield of white solid. ^1^H NMR (400 MHz, Chloroform-*d*) δ 7.16 (d, *J =* 5.9 Hz, 4H), 4.63 (s, 4H), 3.84 (s, 6H), 2.31 (s, 6H). ^13^C NMR (101 MHz, Chloroform-d) δ 168.61, 151.80, 139.11, 129.01, 124.91, 121.20, 116.90, 56.31, 45.70, 20.58.

##### 2.5.4. (Oxybis(methylene))bis(2-bromo-6-methoxy-4,1-phenylene) diacetate (**4b-4**)


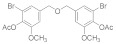


76% yield of white solid. ^1^H NMR (400 MHz, Chloroform-d) δ 7.21 (d, *J =* 1.9 Hz, 2H), 6.94 (d, *J =* 1.9 Hz, 2H), 4.52 (s, 4H), 3.85 (s, 6H), 2.36 (s, 6H). ^13^C NMR (101 MHz, Chloroform-d) δ 167.84, 152.55, 137.89, 136.87, 124.36, 117.21, 111.57, 56.30, 45.19, 20.44.

##### 2.5.5. (Oxybis(methylene))bis(5-bromo-2-methoxy-4,1-phenylene) diacetate (**4b-5**)


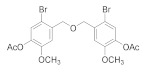


82% yield of white solid. ^1^H NMR (Acetone, 400 MHz) δ 7.39 (1H, s), 7.37 (1H, s), 4.78 (2H, s), 3.86 (3H, s), 2.26 (3H, s). ^13^C NMR (CDCl_3_, 101 MHz) δ 168.59 (H, s), 150.93 (H, s), 140.27 (H, s), 135.04 (H, s), 127.23 (H, s), 114.36 (H, s), 113.56 (H, s), 56.27 (H, s), 46.03 (H, s), 20.65 (H, s).

##### 2.5.6. 2,3-Dibromo-6-methoxy-4-(methoxymethyl) phenol (**4b-6**) 


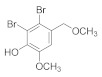


**Scheme 5:**
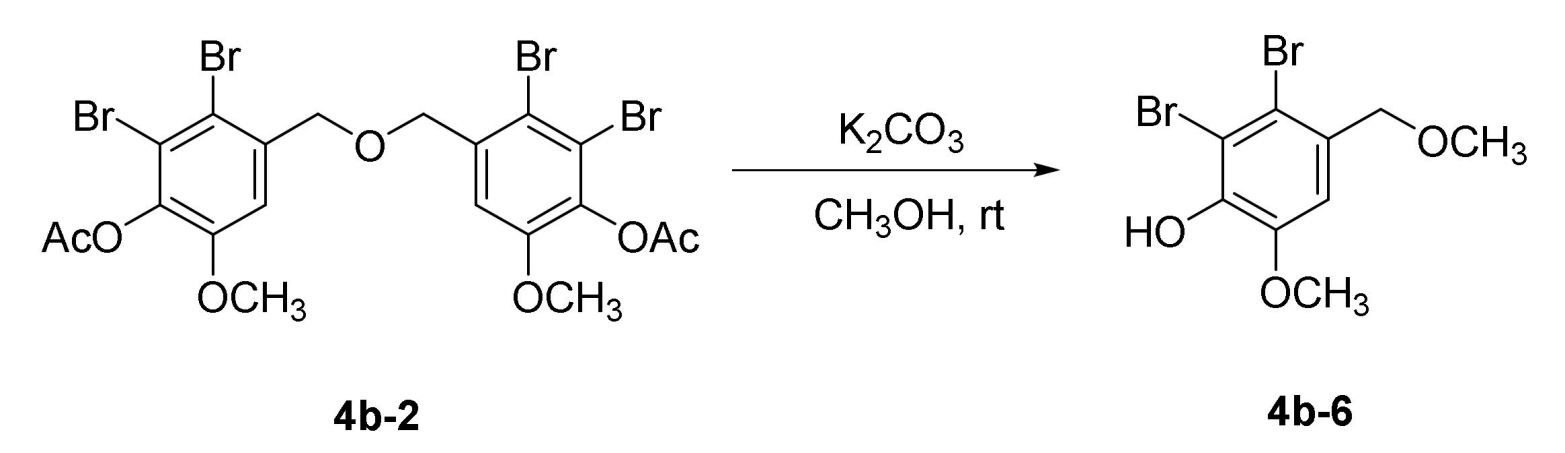
Synthesis of 2,3-dibromo-6-methoxy-4-(methoxymethyl) phenol (**4b-6**).

The compound **4b-2** (69 mg, 0.1 mmol) was added to methanol solution, and the solution became a suspension. Then, potassium carbonate (41 mg, 0.3 mmol) was added and stirred (Scheme 5). When reaction was complete as indicated by TLC, the mixture was filtered through a Celite pad, and the filtrate was concentrated to give the crude product. The crude product was chromatographed on silica gel (Hex/EtOAc) to give the compound **4b-6** (92% yield as white solid). ^1^H NMR (400 MHz, Chloroform-d) δ 7.01 (s, 1H), 6.12 (s, 1H), 4.48 (s, 2H), 3.91 (s, 3H), 3.46 (s, 3H). ^13^C NMR (101 MHz, Chloroform-d) δ 145.94, 143.30, 130.45, 115.47, 111.82, 109.64, 74.56, 58.39, 56.26.

### 2.6. Cell Lines and Cell Culture

The immortalized human keratinocyte cell line HaCaT and the human chronic myelogenous leukemia K562 cells were maintained in dulbecco’s modified eagle’s medium (DMEM) and in cove’s modified dulbecco’s medium (IMDM) (GIBCO, Grand Island, NY, USA), respectively, containing 10% fetal bovine serum (FBS) and 1% penicillin/streptomycin in a humidified incubator at 37 °C with 5% CO_2_.

### 2.7. Cell Viability Assessment

The K562 cell viability was determined by using the 3-(4,5-dimethylthiazol-2-yl)-2,5-diphenyltetrazolium bromide (MTT) method. K562 cells were seeded in 96-well plates and treated with different concentrations of **3b-1** to **9** and **4b-1** to **6** compounds for 24, 48 and 72 h. Then, the MTT (100 μL) was added in each sample and incubated at 37 °C for 4 h. Then, acidic isopropanol (100 μL) was added in each sample and incubated overnight to dissolve the formazan product at 37 °C. Finally, samples’ absorbance was measured at 570 nm using a microplate reader (BioTek, Winooski, VT, USA).

The HaCaT cell viability was determined by sulforhodamine B (SRB) assay method. HaCaT cells were seeded in 96-well plates and cultured overnight. Then, cells were treated with different concentrations of **3b-1** to **9** and **4b-1** to **6** compounds for 24 or 72 h. For the H_2_O_2_ model, cells were treated with H_2_O_2_ (500 µM) for 3 h to induce oxidative stress. After treatment, cells were fixed with 10% trichloroacetic acid (100 µL) at 4 °C for 1 h, then, after rinsing and drying the plates, the cells were incubated with SRB (100 µL) for 15 min. Finally, the bound stain was dissolved with Tris buffer (150 µL) and samples’ absorbance was detected at 515 nm on a microplate reader (BioTek, Winooski, VT, USA). 

### 2.8. Intracellular ROS Assay

HaCaT cells were seeded in a 24-well plate treated with **3b-9** and **4b-3** (5 and 10 µM) for 24 h. After the pretreatment, the cells were further incubated with H_2_O_2_ (500 µM) for 1 h. Then, the cells were incubated with DCFH-DA (10 µM) in serum-free DMEM in the dark for 20 min. After the incubation, the cells were washed with serum-free DMEM, and the fluorescence intensity was detected by fluorescence microscopy (Leica, Wetzlar, Hessen, Germany). Finally, the ROS level was determined by fluorescence level.

### 2.9. Cell Apoptosis Analysis

For the assessment of apoptosis in K562 cell line, cells were seeded in a 6-well plate (1 × 10 ^5^ cells/well) and cultured in the presence of different concentrations of **4b-4** for 24 h.

For HaCaT cell line, cells were seeded in a 6-well plate (1 × 10 ^5^ cells/well) and allowed to adhere overnight. Then, cells were treated with different concentrations of **3b-9** and **4b-3** for 24 h followed by H_2_O_2_ (500 µM) treatment for 1 h. 

After treatment, both K562 and HaCaT cells were collected, centrifuged, washed with phosphate buffered saline (PBS) and stained according to the instructions of the cell apoptosis kit (Absin, Shanghai, China). Finally, the cell apoptosis was analyzed by flow cytometer (CytoFLEX, Beckman Coulter, Suzhou, China).

### 2.10. Cell Cycle Analysis

K562 cells were seeded in a 6-well plate (1 × 10 ^5^ cells/well) and cultured in the presence of different concentrations of **4b-4** for 24 h. After incubation, K562 cells were collected, centrifuged, washed with PBS and fixed with 70% ethanol overnight at −20 °C. Then, the cells were centrifuged to remove ethanol washed twice with PBS and stained with propidium iodide (PI) for 30 min at 37 °C in the dark according to the instructions of the Cell Cycle and Apoptosis Analysis Kit (Beyotime, Shanghai, China). The cell cycle was analyzed by flow cytometer (CytoFLEX, Beckman Coulter, Suzhou, China) at emission wavelength of 488 nm.

### 2.11. Western Blotting Assay

HaCaT cells were seeded in 6-well plates (1 × 10 ^5^cells/well). Then, cells were treated with different concentration (2.5–10 µM) of **3b-9** or **4b-3** for 24 h. Cells were collected, washed, and lysed. Then, the cell lysate was boiled, separated by electrophoresis on sodium dodecyl sulfate polyAcrylamide gel electrophoresis (SDS-PAGE, 6–12%), and transferred to nitrocellulose filter membranes (Millipore, Billerica, MA, USA). The nitrocellulose filter membranes were blocked and incubated with the primary antibodies. Subsequently, the membranes were incubated with HRP-secondary antibody and detected by Tanon 5200 (Tanon, Beijing, China). In addition, antibodies against HO-1, TrxR1, Nrf2 and Keap1 were purchased from Cell Signaling Technology (Boston, MA, USA). Antibody against GAPDH was obtained from Huaan Biotechnology Co., Ltd. (Hangzhou, China).

### 2.12. Statistical Analysis

The results shown in this study were represented as the mean ± SD. Comparisons between the groups were assessed by one-way analysis of variance (ANOVA), and a multiple comparison test was performed using the post hoc Bonferroni correction. *p* < 0.05 was defined as statistically significant.

## 3. Results and Discussion

### 3.1. Chemical Synthesis

We have synthesized a series of methylated (**3b-1** to **9**) and acetylated (**4b-1** to **5**) derivatives of natural bromophenols, in order to evaluate their anticancer and antioxidant activities. 3-bromo-4-hydroxy-5-methoxybenzaldehyde (**1b**) was synthesized by monohalogenation of bromine with vanillin from commercial sources in the presence of acetic acid.

On this basis, the dihalogenated product 2,3-dibromo-4-hydroxy-5-methoxybenzaldehyde (**1c**) was obtained by adding iron powder as catalyst under reflux condition or by halogenation with iron powder catalyzed in buffer solution of acetic acid and sodium acetate get 2-bromo-3-hydroxy-4-methoxybenzaldehyde (**2b**). These products, together with some commercial bromobenzaldehyde, were then methylated with dimethyl sulfate, reduced with aldehyde group with sodium borohydride and finally methylated with dimer (**3b-1** to **9**) with triethylamine and MsCl. Moreover, in the condition of pyridine and acetic anhydride, they were exposed to acetylation, followed by reduction and dimerization to obtain acetylation of dimer products (**4b-1** to **5**). Methylation and acetylation of bromophenol compounds are easier prepared and exhibit greater stability compared to bromophenol compounds having hydroxyl groups completely exposed. The ^1^H NMR, ^13^C NMR, HRMS, and HPLC spectra of compound **3b-1** to **9** and **4b-1** to **6** are provided in Supplementary Figures S1–S40.

### 3.2. The Cellular Antioxidant Activity of **3b-1** to **9** and **4b-1** to **6**

#### 3.2.1. **3b-9** and **4b-3** Ameliorates H_2_O_2_-Induced Oxidative Cell Damage

**Figure 2:**
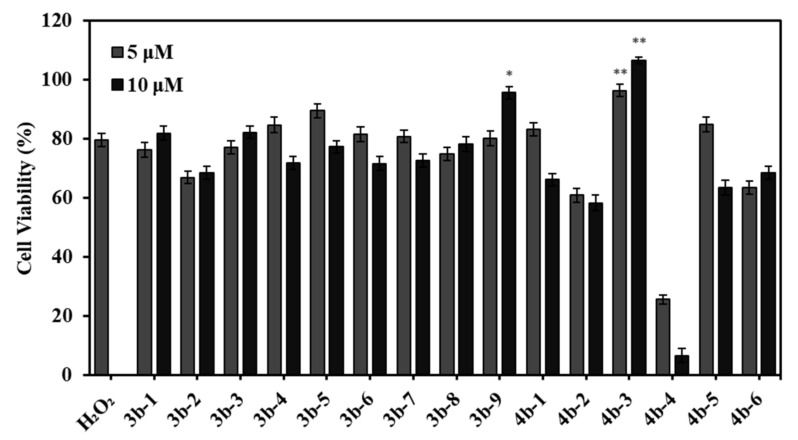
**3b-9** and **4b-3** reverses oxidative damage in HaCaT cells. HaCaT cells were incubated with different concentration of **3b-9** and **4b-3** (5 and 10 µM) for 24 h and then were treated with H_2_O_2_ for 3 h. The cell viability was determined by the SRB method. Values are expressed as mean ± SD of three independent experiments. * *p* < 0.05, ** *p* < 0.01, versus H_2_O_2_ group.

First, we detected the antioxidant activity of **3b-1** to **9** and **4b-1** to **6** at the cellular level using H_2_O_2_-induced oxidative damage model in skin HaCaT cells. As shown in Figure 2, H_2_O_2_ could significantly reduce HaCaT cell viability, among the tested compounds, **4b-2** and **4b-4** exacerbated H_2_O_2_-induced cell oxidative damage, **3b-1** to **3b-8**, **4b-1**, **4b-2** and **4b-4** to **4b-6** had no significant effect on H_2_O_2_-induced cellular oxidative damage, while **3b-9** and **4b-3** could ameliorate H_2_O_2_-induced oxidative cell damage indicated that these compounds had antioxidant effects at the cellular level.

**Figure 3:**
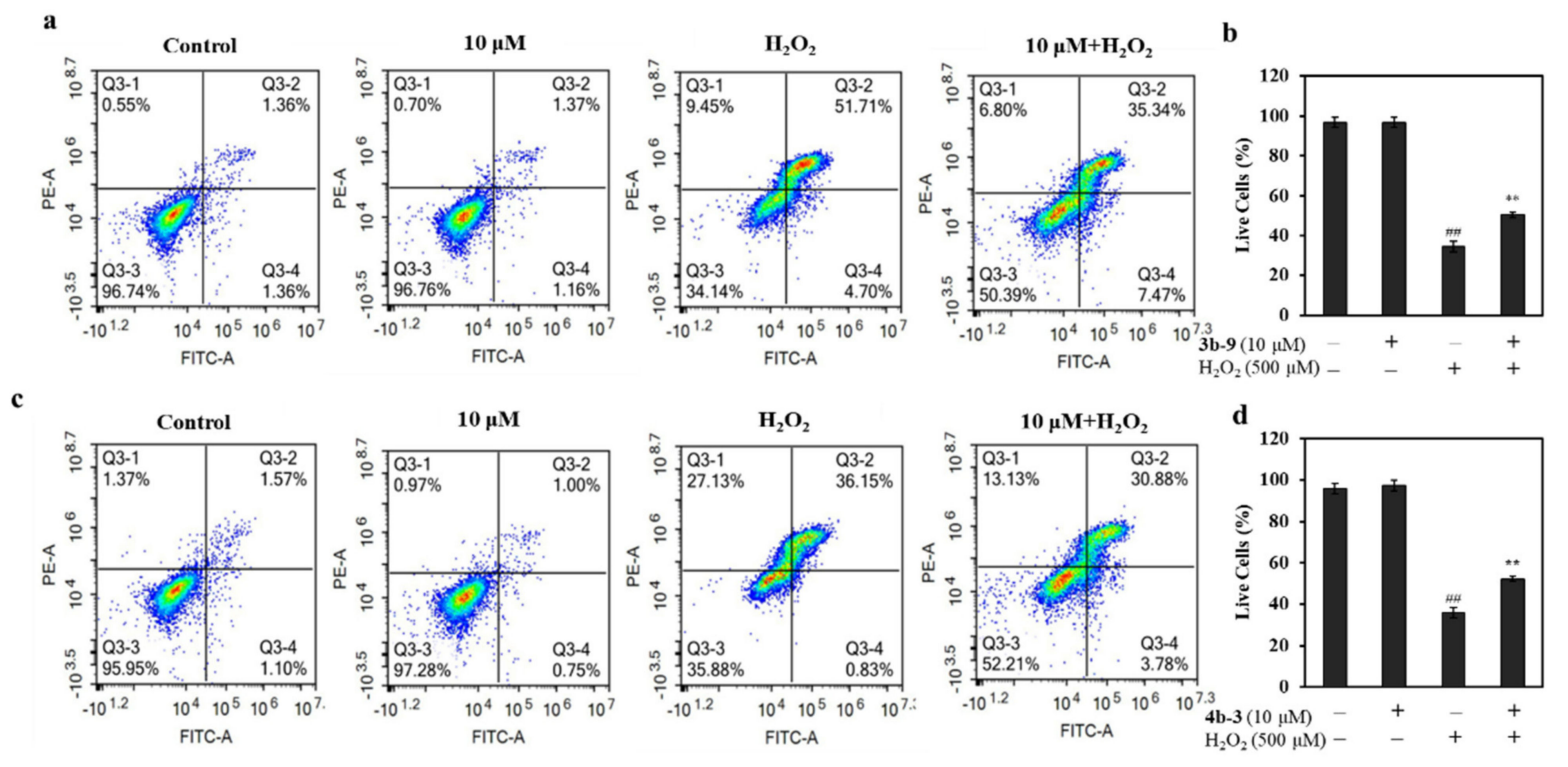
**3b-9** and **4b-3** alleviated H_2_O_2_-induced HaCaT cell damage. (**a**) In the presence or absence of H_2_O_2_, the ratio of apoptosis and viability of HaCaT cells treated with **3b-9** (10 μM) were measured by flow cytometry. (**b**) The histogram depicted the proportion of living HaCaT cells after **3b-9** treatment. (**c**) In the presence or absence of H_2_O_2_, the ratio of apoptosis and viability of HaCaT cells treated with **4b-3** (10 μM) were measured by flow cytometry. (**d**) The histogram depicted the proportion of living HaCaT cells after **4b-3** treatment. ^##^ *p* < 0.01, versus control group; ** *p* < 0.01, versus H_2_O_2_ group.

To further investigate the protective activity of **3b-9** and **4b-3** on oxidative damaged cells, their effects on H_2_O_2_ induced-apoptosis in HaCaT cells were assessed by flow cytometry using Annexin V/PI double staining method. As shown in Figure 3, **3b-9** and **4b-3** treatment alone did not induce HaCaT cell damage at the concentration of 10 µM. On the other hand, H_2_O_2_ treatment decreased cell viability and increased apoptosis of HaCaT cells. However, the live cell rate increased from 34.14 to 50.39% and from 35.88 to 52.21 after pretreatment with **3b-9** and **4b-3** (10 μΜ), respectively, followed by the presence of H_2_O_2_ (Figure 3). Thus, these results indicated that **3b-9** and **4b-3** exerted antioxidant activity at cellular level.

#### 3.2.2. **3b-9**and **4b-3** Decreases the H_2_O_2_-Induced ROS Generation in HaCaT Cells

**Figure 4:**
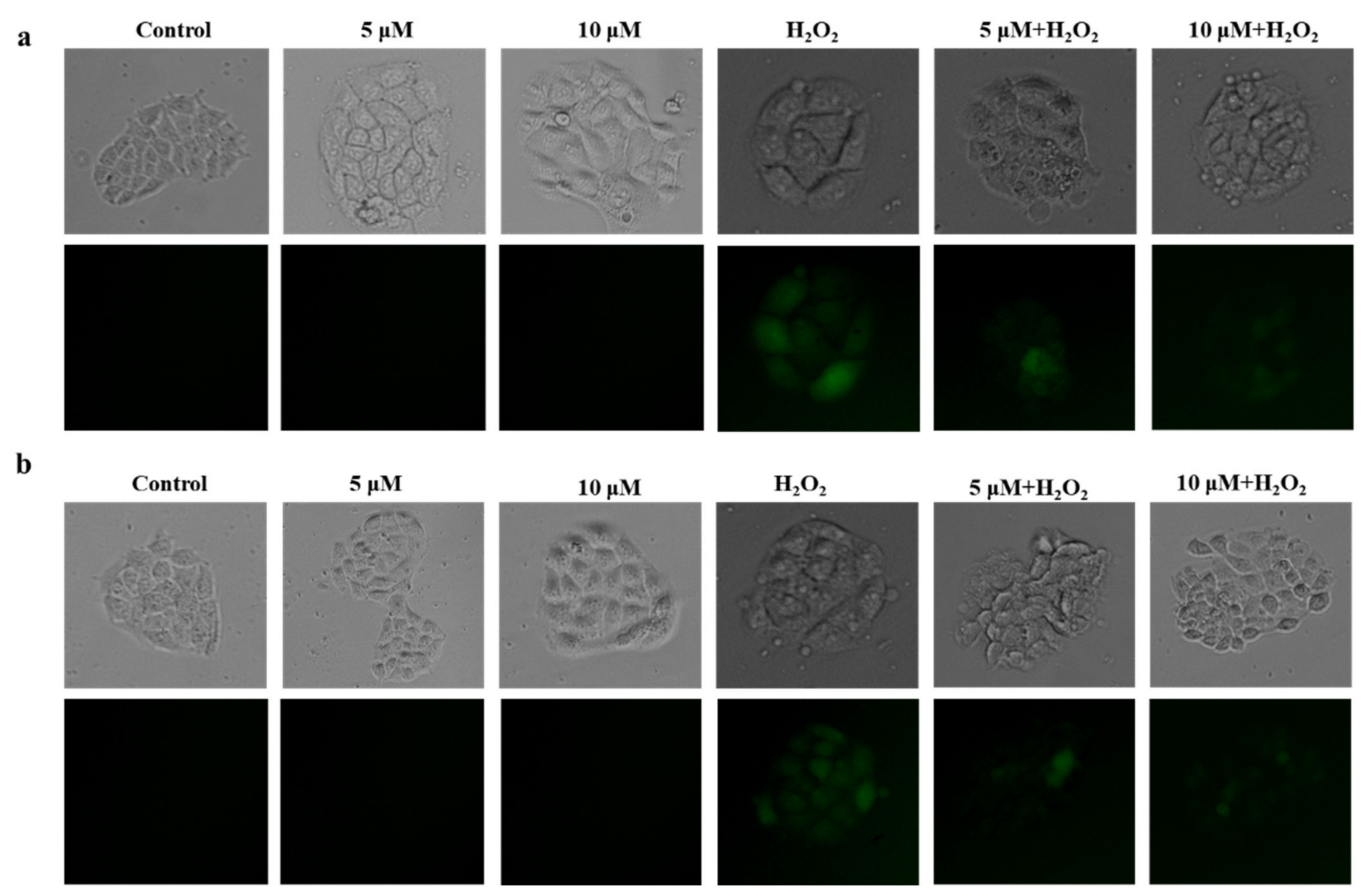
**3b-9** and **4b-3** reduces the ROS level in HaCaT cells. HaCaT cells were pretreated with **3b-9** (**a**) and **4b-3** (**b**) for 24 h and then were exposed to H_2_O_2_ (500 μM) for 1 h. Cells were incubated with DCFH-DA (10 μM) for 20 min, and the ROS level were observed with fluorescence microscopy.

The excessive generation of ROS is related with oxidative damage to the HaCaT cells. Next, we explored the effect **3b-9** and **4b-3** on the ROS level in HaCaT cells. As shown in Figure 4, H_2_O_2_ could significantly increase the ROS level in HaCaT cells and **3b-9** (Figure 4a) and **4b-3** (Figure 4b) alone did not affect the level of ROS in HaCaT cells. However, after **3b-9** and **4b-3** pretreatment, we found that **3b-9** and **4b-3** could significantly decrease H_2_O_2_-induced ROS generation in HaCaT cells (Figure 4). This result further indicated that **3b-9** and **4b-3** has antioxidant activity at the cellular level.

#### 3.2.3. **3b-9**and **4b-3** Promotes the Expression of the Antioxidant Protein TrxR1 and HO-1

**Figure 5:**
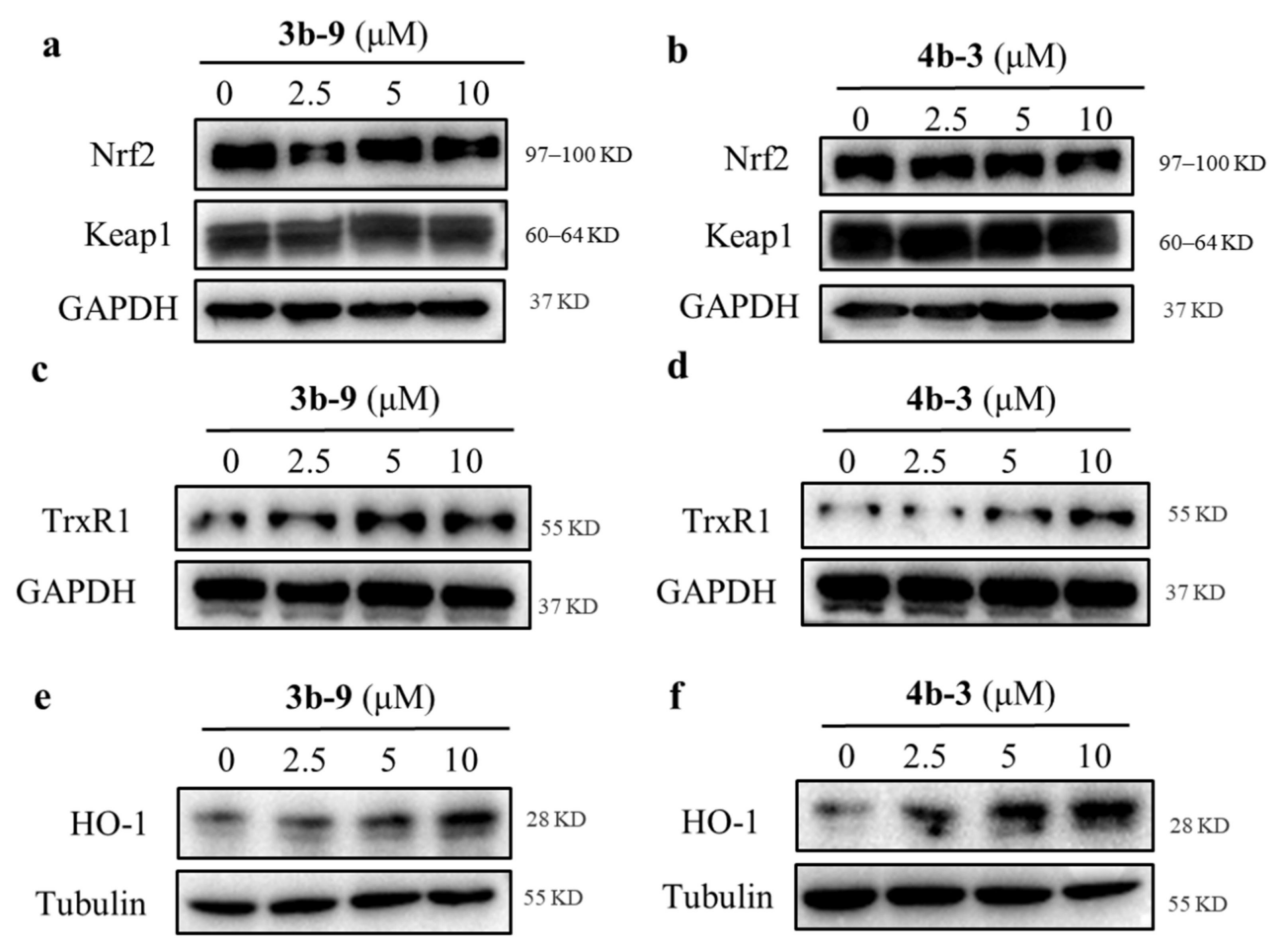
The effect of **3b-9** and **4b-3** on the expression of Nrf2, Keap1, TrxR1 and HO-1. (**a**) HaCaT cells were treated with **3b-9** (2.5–10 µM) for 24 h. The expression of Nrf2 and Keap1 was detected by Western blotting assay. (**b**) HaCaT cells were treated with **4b-3** (2.5–10 µM) for 24 h. The expression of Nrf2 and Keap1 was detected by Western blotting assay. (**c**) HaCaT cells were treated with **3b-9** (2.5–10 µM) for 24 h. The expression of TrxR1 was detected by Western blotting assay. (**d**) HaCaT cells were treated with **4b-3** (2.5–10 µM) for 24 h. The expression of TrxR1 was detected by Western blotting assay. (**e**) HaCaT cells were treated with **3b-9** (2.5–10 µM) for 24 h. The expression of HO-1 was detected by Western blotting assay. (**f**) HaCaT cells were treated with **4b-3** (2.5–10 µM) for 24 h. The expression of HO-1 was detected by Western blotting assay. All the original images for the Western blotting assay are provided in Supplementary Figures S41–S46.

Next, we explored the molecular mechanism of **3b-9** and **4b-3** exerting antioxidant effects in HaCaT cells. First, we tested the effect of **3b-9** and **4b-3** on the Nrf2 signaling pathway, which plays an important role in the cellular oxidative stress. As shown in Figure 5a,b, under the present experimental conditions, **3b-9**and **4b-3** did not affect the expression of Nrf2 and Keap1, which indicated that **3b-9** and **4b-3** exert antioxidant effect was not through modulating the expression level of Nrf2 or Keap1. This observation was different from some of the natural bromophenols which could increase the level of Nrf2 [18] and decrease the level of Keap1 [6,19]. For example, marine bromophenol bis (2,3,6-T ribromo-4,5-dihydroxybenzyl) ether could protect HaCaT skin cells from oxidative damage by increasing Nrf2 and decreasing Keap1 expression [6].

However, we found that both **3b-9** and **4b-3** could increase the expression of the antioxidant protein TrxR1 and HO-1 (Figure 5c-f), which was the downstream proteins of Nrf2 pathway [6] and NFκB pathway [20]. Considering that **3b-9** and **4b-3** did not affect the expression level of NrF2, we deduced that **3b-9** and **4b-3** increasing the expression of TrxR1 was not through the Nrf2 expression, while possibly via other Nrf2-independent pathways. Furthermore, studies have revealed that HO-1 was not only activated by Nrf2, but also by AMPK signaling pathway [21]. Therefore, we speculated that the increased expression of HO-1 may be related to the AMPK signaling pathway, and the antioxidant effects of compound **3b-9** and **4b-3** in HaCaT cells indicate their potential application in the oxidative damage related diseases, such as aging and diabetic complications.

### 3.3. Cytotoxicity of **3b-1** to **9** and **4b-1** to **6** against Cancer Cells

To examining the potential anticancer activity of these synthesized compounds, their ability to inhibit K562 and HaCaT cell survival was assessed. Among these tested compounds, **4b-4**, **3b-6**, **3b-8**, **3b-9** and **4b-5** inhibited the viability of K562 cells (Table 1). Specifically, **4b-4** exhibited the strongest inhibitory effect and reduced K562 cell viability to 35.27% at a concentration of 10 µM. Furthermore, **3b-8**, a derivative of **3b-1**, inhibited K562 cell growth, while **3b-1** had no effect (Table 1). This result indicated that the addition of Br group played an important role in **3b-8** compound’s antiproliferative effect.

Regarding the tested compounds’ effects on HaCaT cell survival, only **4b-4** reduced the viability of HaCaT cells, namely to 4.19% at the concentration of 10 µM (Table 1). Furthermore, **4b-4** could significantly reduce the cell viability of K562 cells rather than the HaCaT cells at the concentration of 5 µM. These results suggesting that **4b-4** had less toxic to normal HaCaT cells and had good selectivity in inhibiting the cell viability of K562 cells.

**Figure 6:**
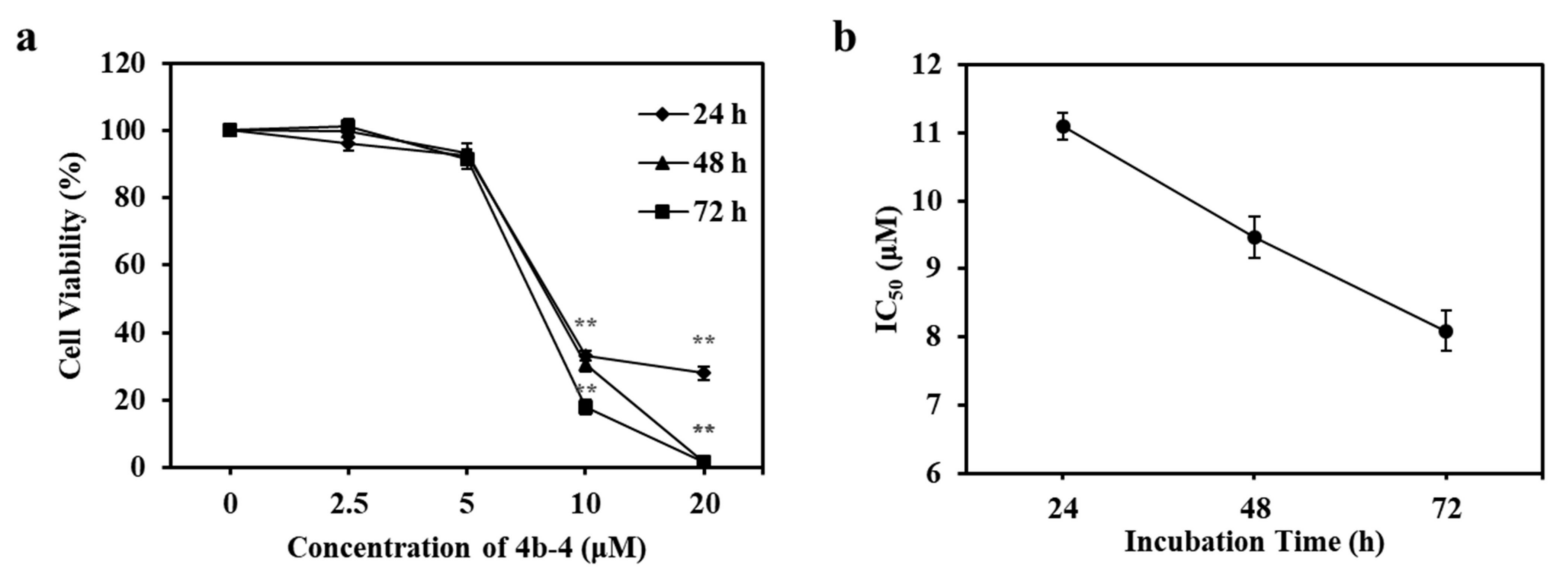
**4b-4** inhibits the viability of K562 cells. (**a**) K562 cells were incubated with different concentration of **4b-4** (2.5–10 µM) for 24, 48 and 72 h. Then, cell viability (%) was assessed by MTT assay. (**b**) IC_50_ values of **4b-4** against K562 cells at 24, 48 and 72 h. Data are presented as mean ± SD for three independent experiments. ** *p* < 0.01.

Since **4b-4** compound exhibited the greatest inhibition against K562 cell survival, its molecular mechanism accounting for this anticancer effect was further investigated. As shown in Figure 6a, **4b-4** inhibited time-dependently the viability of K562 cells after 24, 48 and 72 h treatment. The IC_50_ values of **4b-4** against K562 cell survival were 11.09, 9.46, and 8.09 µM, at 24, 48, and 72 h, respectively. 

**Figure 7:**
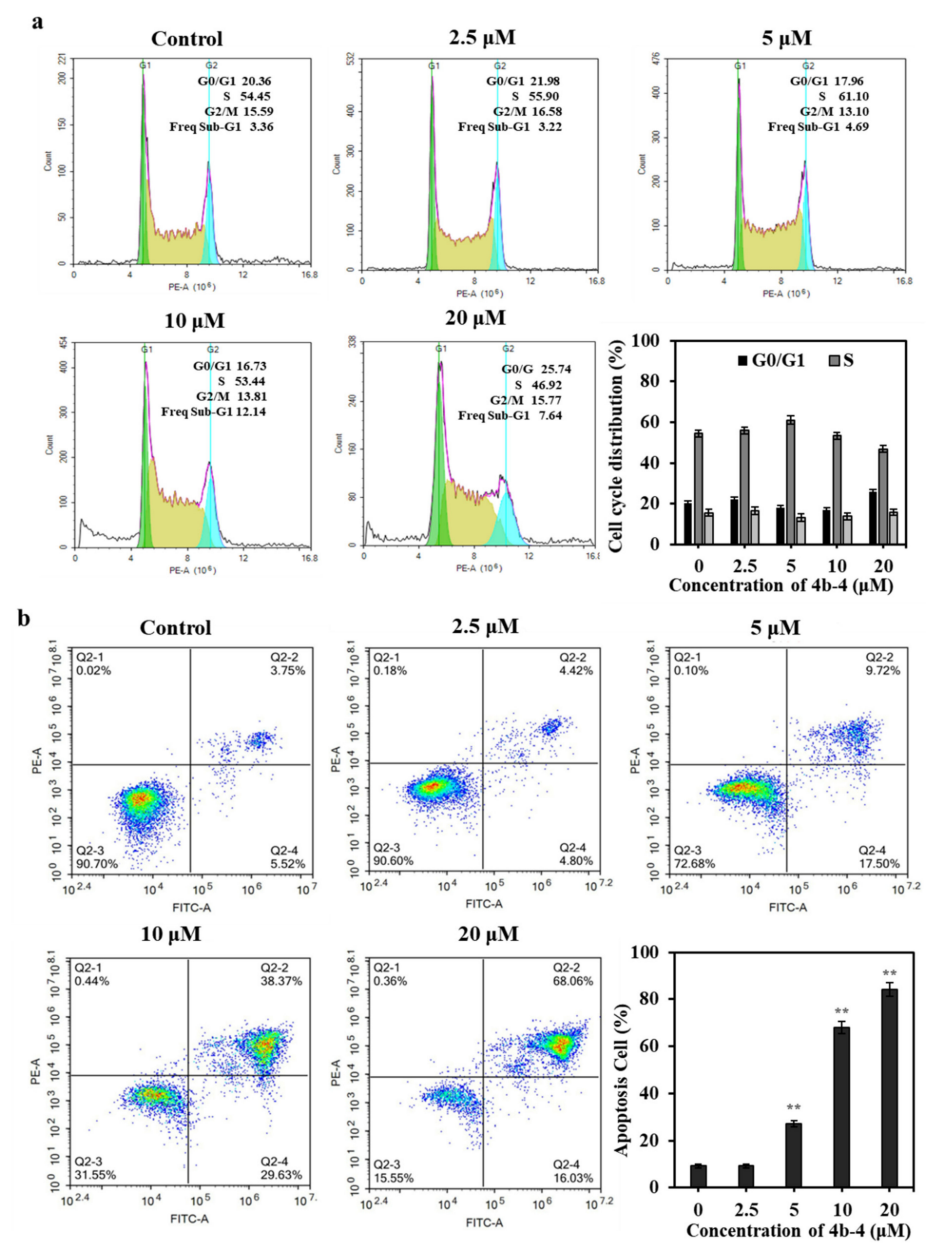
Assessment of apoptosis and cell cycle arrest in K562 cells treated with **4b-4.** (**a**) K562 cells were treated with **4b-4** (2.5–20 µM) for 24 h, and then cell cycle was analyzed by flow cytometer. The histogram shows the proportion of each cell cycle phase. (**b**) K562 cells were treated with **4b-4** (2.5–20 µM) for 24 h, and then cell apoptosis was analyzed by flow cytometry. The histogram shows the percentage of apoptotic cells. Data are presented as mean ± SD for three independent experiments. ** *p* < 0.01.

To further investigate the mechanism underlying **4b-4** inhibition against K562 cell viability, cell cycle arrest assay was performed by flow cytometry after PI staining. As shown in Figure 7a, **4b-4** had no significant effect on any cell phase (i.e., G0/G1, S, and G2/M) compared to control. However, the appearance of sub-G0/G1 phase prompted us to examine whether **4b-4** could induce apoptosis in K562 cells. Therefore, we then detected whether **4b-4**-induced apoptosis in K562 cells. As shown in Figure 7b, using Annexin V/PI double staining method, the proportion of apoptotic cells was increased from 3.75% in control cells to 27.22, 68.00, and 84.09% in cells treated with **4b-4** at 5, 10, and 20 µM, respectively. Thus, these results indicated that **4b-4** inhibited K562 cell viability by inducing apoptosis, and their potential in vivo anticancer activity will be investigated in our future work. Furthermore, their possible toxicity in vivo should be kept in mind and evaluated in details since some polybrominated diphenyl ethers, which are structurally to these synthesized derivatives, are suspected to show negative impact on human and animal health [22,23].

## 4. Conclusions

In summary, we synthesized a series of new natural bromophenol derivatives from easily available materials via simple operation procedures, and all of them have been evaluated on antioxidant and anticancer activities. In particular, the results demonstrated that compounds **3b-9** and **4b-3** were shown to ameliorate H_2_O_2_-induced oxidative damage in HaCaT keratinocyte cells. Mechanism studies show that **3b-9** and **4b-3** could decrease the ROS level and increase the expression of antioxidant protein TrxR1 and HO-1 while not affect Nrf2. Finally, **4b-4** inhibited viability and induced apoptosis of leukemia K562 cells, suggesting its potential anticancer activity.

## Figures and Tables

**Table 1:** The effect of **3b-1** to **9** and **4b-1** to **6** on the viability of K562 and HaCaT cells.

Number	Concentration (μM)	K562 Cell Viability (%)	HaCaT Cell Viability (%)
**3b-** **1**	5	91.35 ± 1.39	97.35 ± 3.48
	10	88.12 ± 0.31	104.96 ± 1.89
**3b-** **2**	5	101.46 ± 1.89	86.50 ± 3.23
	10	101.87 ± 1.08	85.35 ± 3.42
**3b-** **3**	5	92.67 ± 2.58	101.43 ± 2.83
	10	96.28 ± 0.92	87.34 ± 4.68
**3b-** **4**	5	102.63 ± 1.54	89.70 ± 4.90
	10	102.83 ± 2.41	91.86 ± 3.82
**3b-** **5**	5	91.32 ± 3.09	106.41 ± 4.15
	10	85.75 ± 0.73	99.59 ± 3.69
**3b-** **6**	5	86.91 ± 0.47	93.14 ± 7.07
	10	74.34 ± 0.95 *	96.67 ± 3.28
**3b-** **7**	5	95.94 ± 2.35	122.03 ± 2.74
	10	85.23 ± 0.20	106.34 ± 2.59
**3b-** **8**	5	73.69 ± 0.91 *	97.21 ± 4.24
	10	49.43 ± 0.92 *	96.50 ± 5.35
**3b-** **9**	5	86.86 ± 2.47	93.63 ± 4.69
	10	78.71 ± 1.22 *	105.39 ± 4.16
**4b-** **1**	5	102.16 ± 5.04	100.47 ± 0.91
	10	99.10 ± 3.23	97.30 ± 3.27
**4b-** **2**	5	98.45 ± 1.82	99.27 ± 0.81
	10	93.38 ± 1.82	92.09 ± 1.75
**4b-** **3**	5	96.43 ± 0.99	94.56 ± 6.84
	10	95.62 ± 1.97	100.07 ± 3.25
**4b-** **4**	5	40.96 ± 3.72 *	100.42 ± 4.27
	10	35.27 ± 0.65 *	4.19 ± 0.21 *
**4b-** **5**	5	90.49 ± 2.17	91.44 ± 2.12
	10	63.93 ± 4.12 *	94.56 ± 6.84
**4b-** **6**	5	98.45 ± 1.82	88.65 ± 4.01
	10	93.38 ± 1.32	84.45 ± 3.25
Adriamycin	1	11.24 ± 0.67 *	3.39 ± 0.08 *

* The cell viability is significantly reduced.

## Data Availability

Data is contained within the article.

## Supplementary Materials

The following supporting information of ^1^H NMR, ^13^C NMR, HRMS, HPLC spectrum and original images for Western blotting can be downloaded at: https://www.mdpi.com/article/10.3390/antiox11040786/s1, Figure S1–S40: The ^1^H NMR, ^13^C NMR, HRMS and HPLC spectrum of compound **3b-1** to **9** and **4b-1** to **6**; Figure S41–S46: The original images for blots of Figure 5.
