# Supplementary material for: Antioxidant and Anticancer Activities of Synthesized Methylated and Acetylated Derivatives of Natural Bromophenols
Source: Antioxidants (Basel). 2022 Apr 15;11(4):786. doi: 10.3390/antiox11040786 (PMC9032154; doi:10.3390/antiox11040786)
Supplement: Supplementary file 1 [file antioxidants-11-00786-s001.zip › antioxidants-1672728Suppl.pdf]

antioxidants

Antioxidant and anticancer activities of synthesized methylated and acetylated derivatives of natural bromophenols

Hui Dong [1,2,#], Li Wang [1,#], Meng Guo[1,2], Dimitrios Stagos [3], Antonis Giakountis [3], Varvara Trachana [4], Xiukun Lin [5], Yankai Liu [1,2,*] and Ming Lriu [1,2,*]

The ^{1}H NMR, ^{13}C NMR, HRMS and HPLC Spectrum of Compound **3b-1** to **9** and **4b-1** to **6**

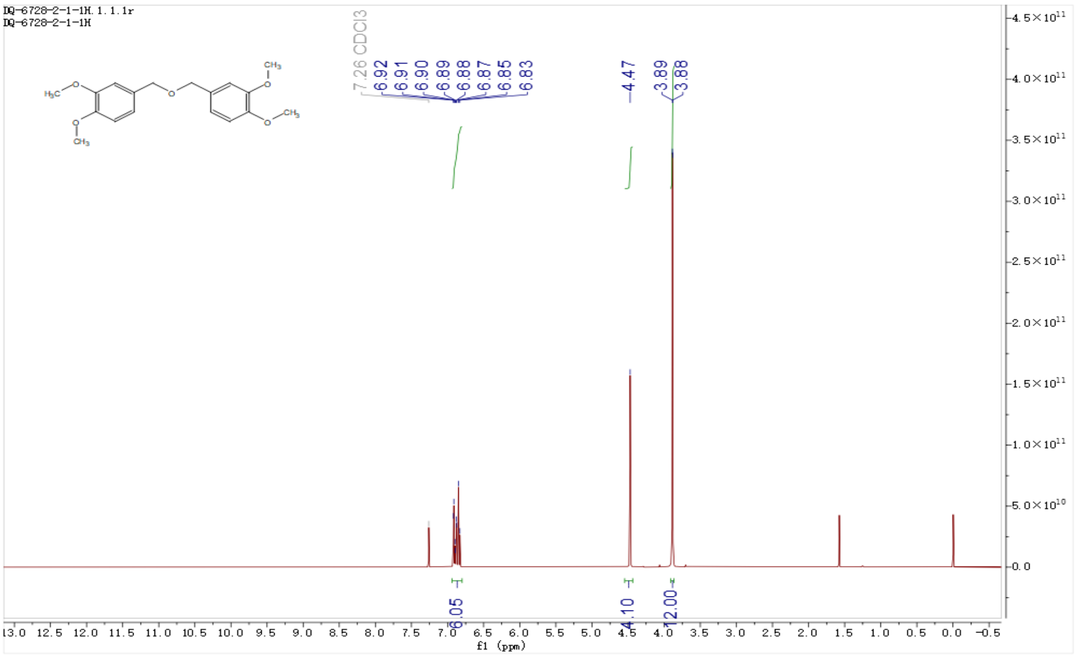

Figure S1. ^{1}H NMR spectrum of compound **3b-1** in Chloroform-*d* (400 MHz).

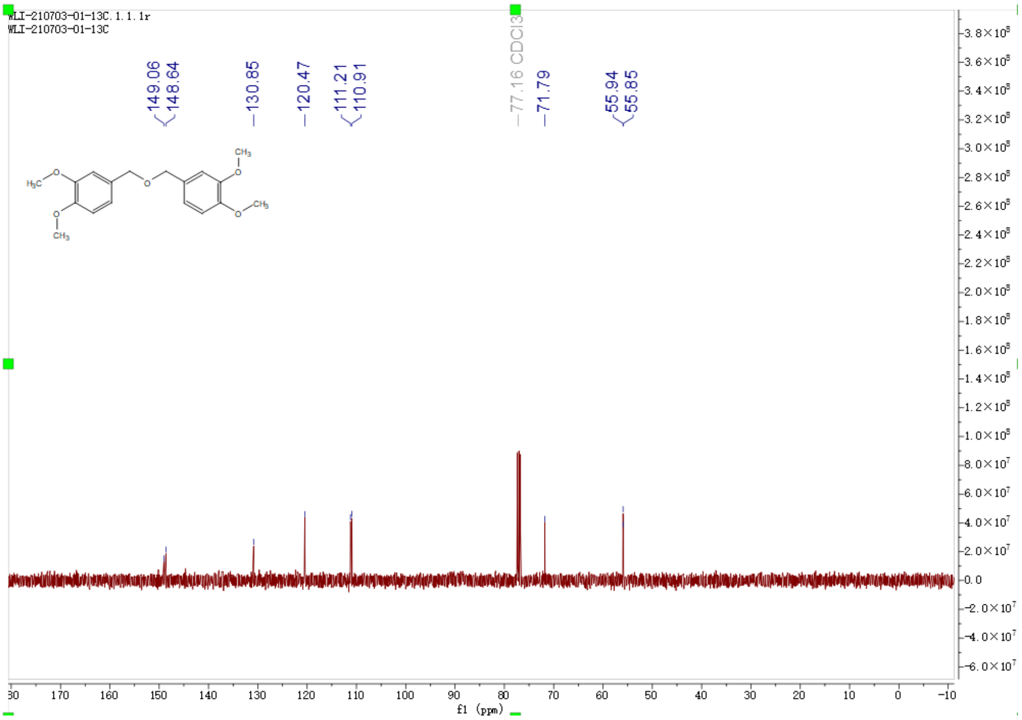

Figure S2. ^{13}C NMR spectrum of compound **3b-1** in Chloroform-*d* (101 MHz).

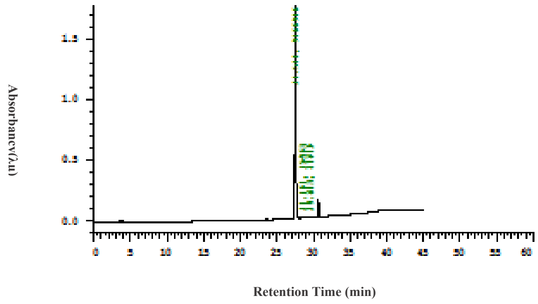

No.	RT	Area	Concentration	BC
1	27.500	9466946	98.917	BV
2	28.460	45963	0.480	VV
3	28.740	22543	0.236	VB
4	29.313	20306	0.212	BV
5	29.673	14842	0.155	VB
		9570600	100.000	

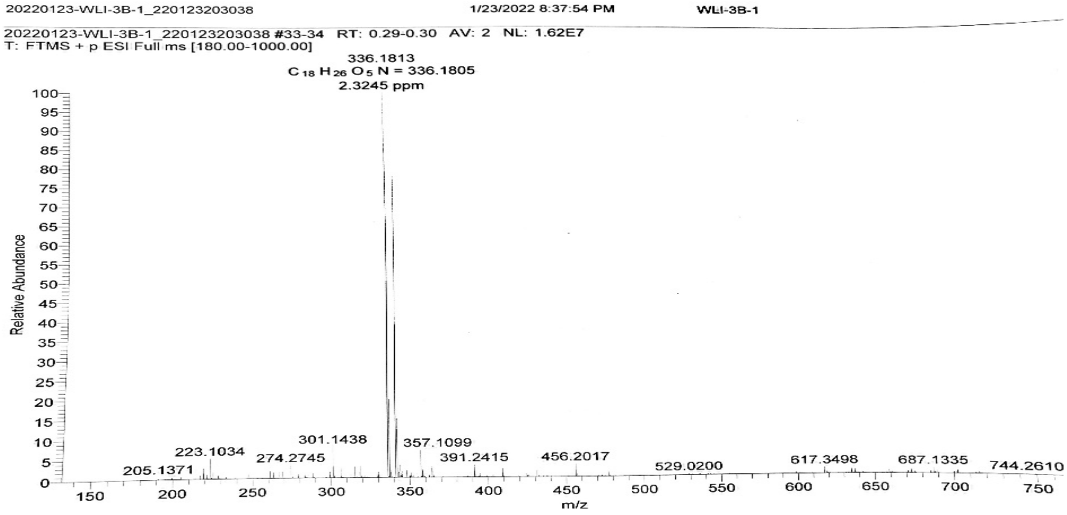

Figure S3. HRMS and HPLC Spectra of **3b-1.**

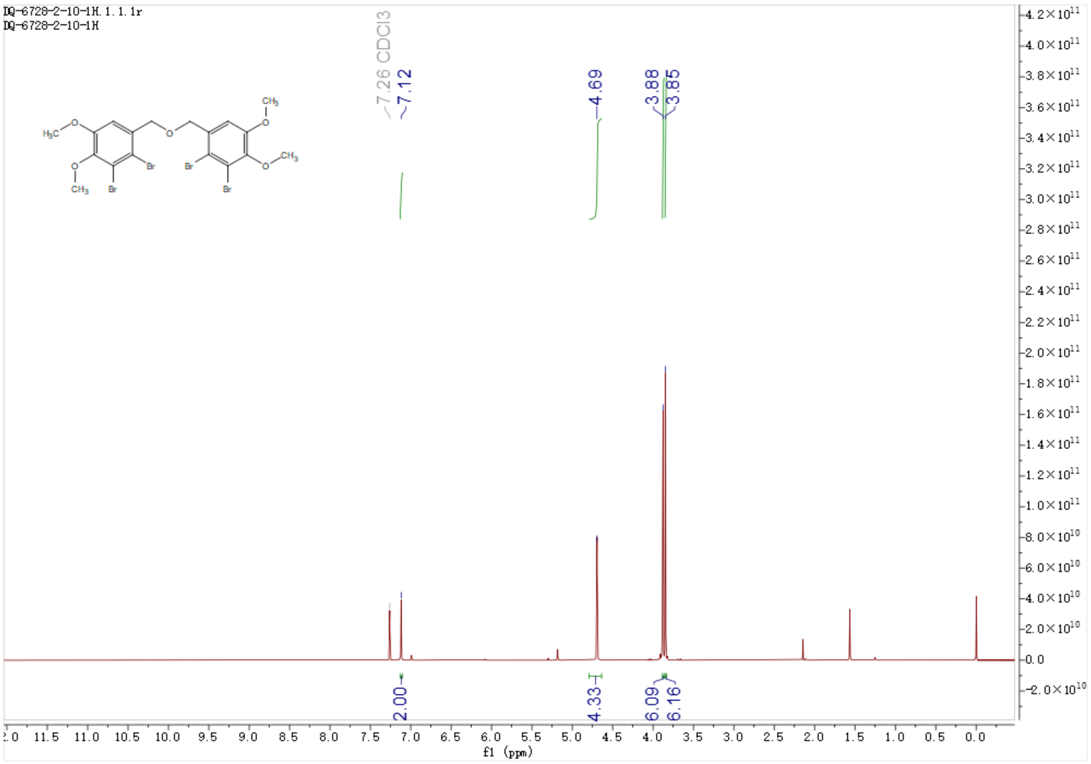

Figure S4. ^{1}H NMR spectrum of compound **3b-2** in Chloroform-*d* (400 MHz).

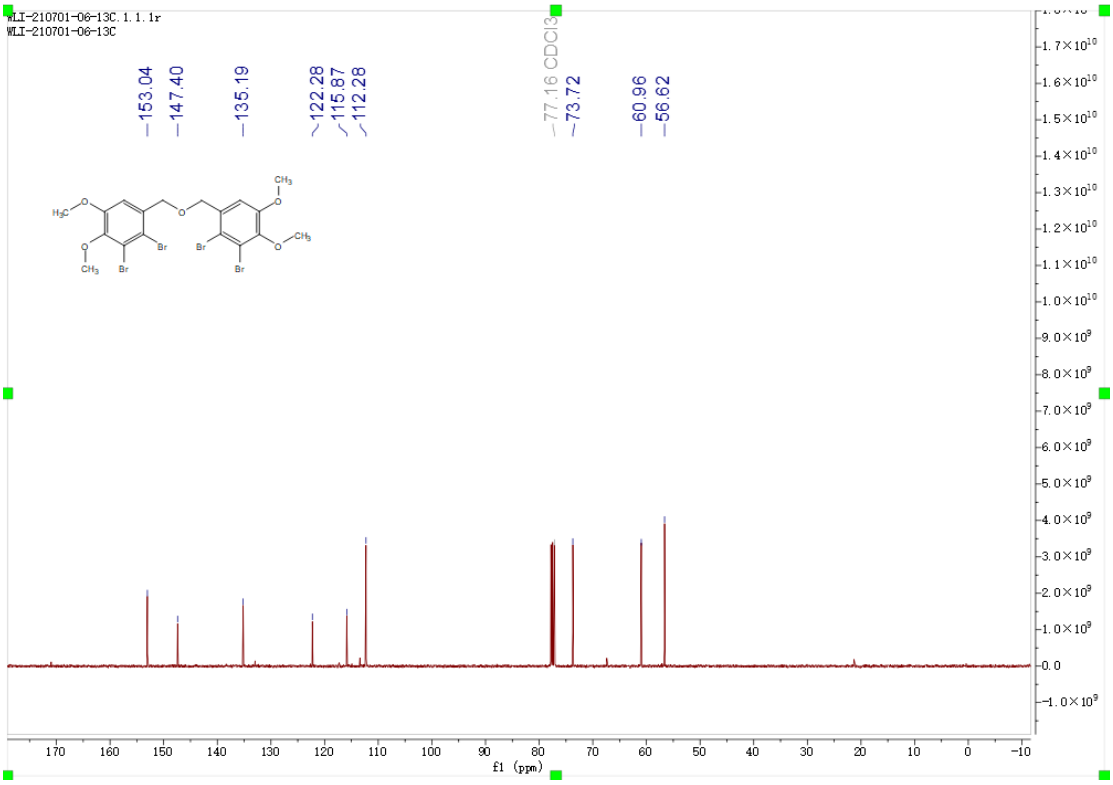

Figure S5. ^{13}C NMR spectrum of compound **3b-2** in Chloroform-*d* (101 MHz).

antioxidants

MDPI

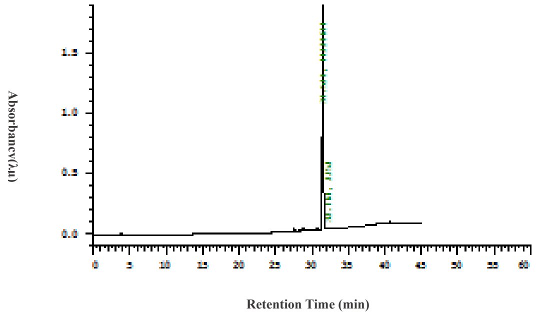

No.	RT	Area	Concentration	BC
1	31.527	10220611	99.978	BB
2	32.067	2252	0.022	BB
		10222863	100.000	

Figure S6. HPLC Spectra of **3b-2.**

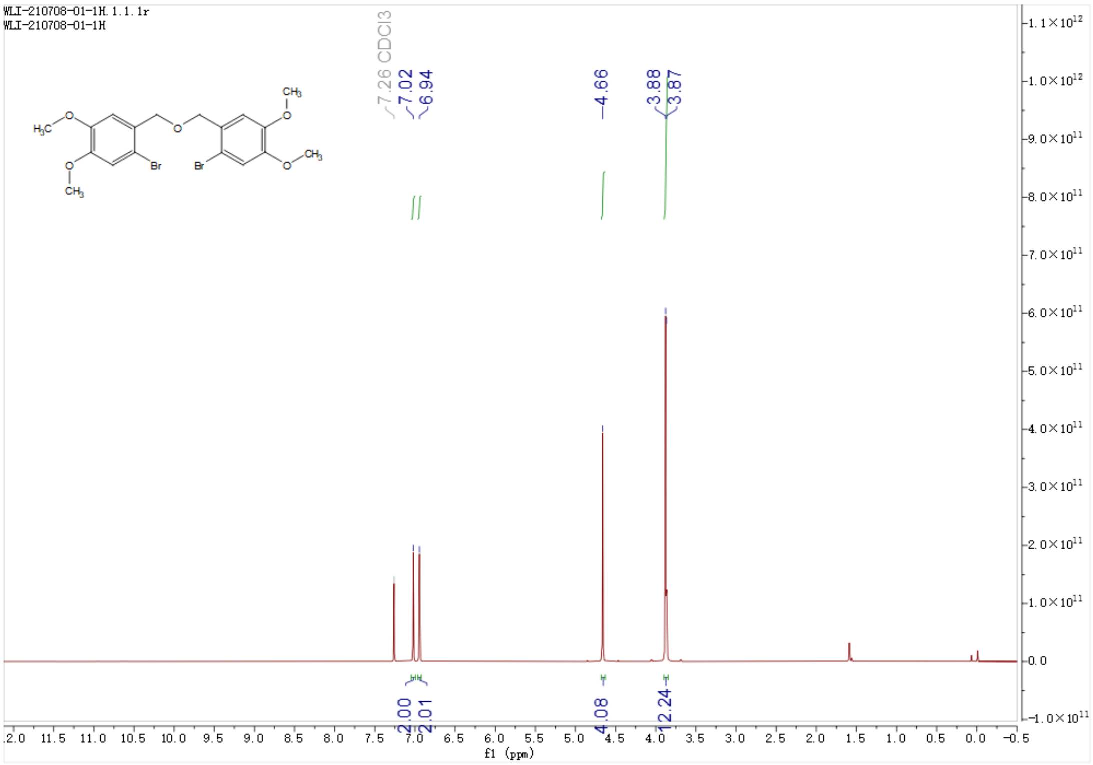

Figure S7. ^{1}H NMR spectrum of compound **3b-3** in Chloroform-*d* (400 MHz).

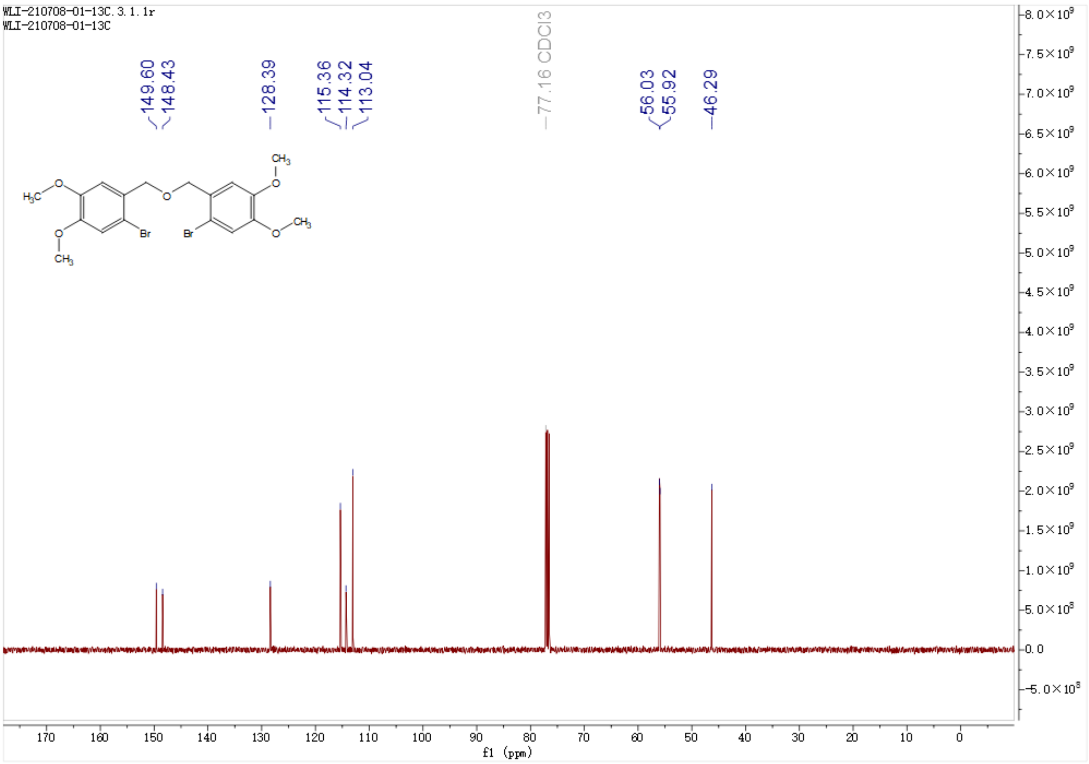

Figure S8. ^{13}C NMR spectrum of compound **3b-3** in Chloroform-*d* (101 MHz).

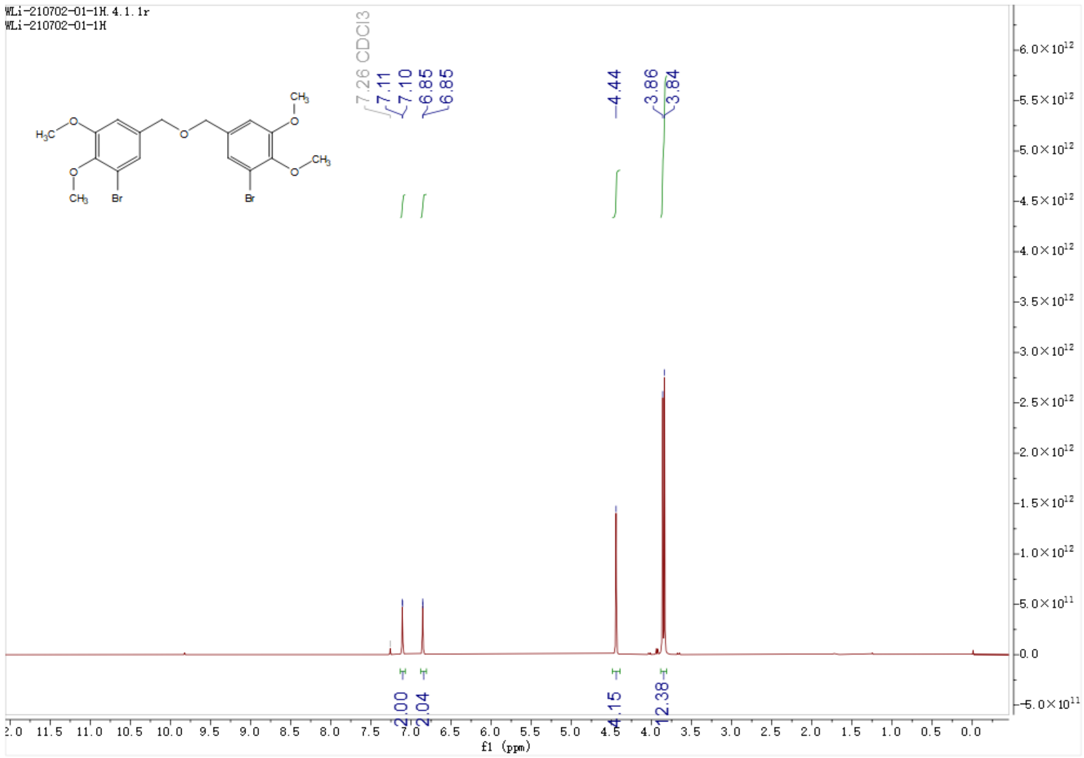

Figure S9. ^{1}H NMR spectrum of compound **3b-4** in Chloroform-*d* (400 MHz).

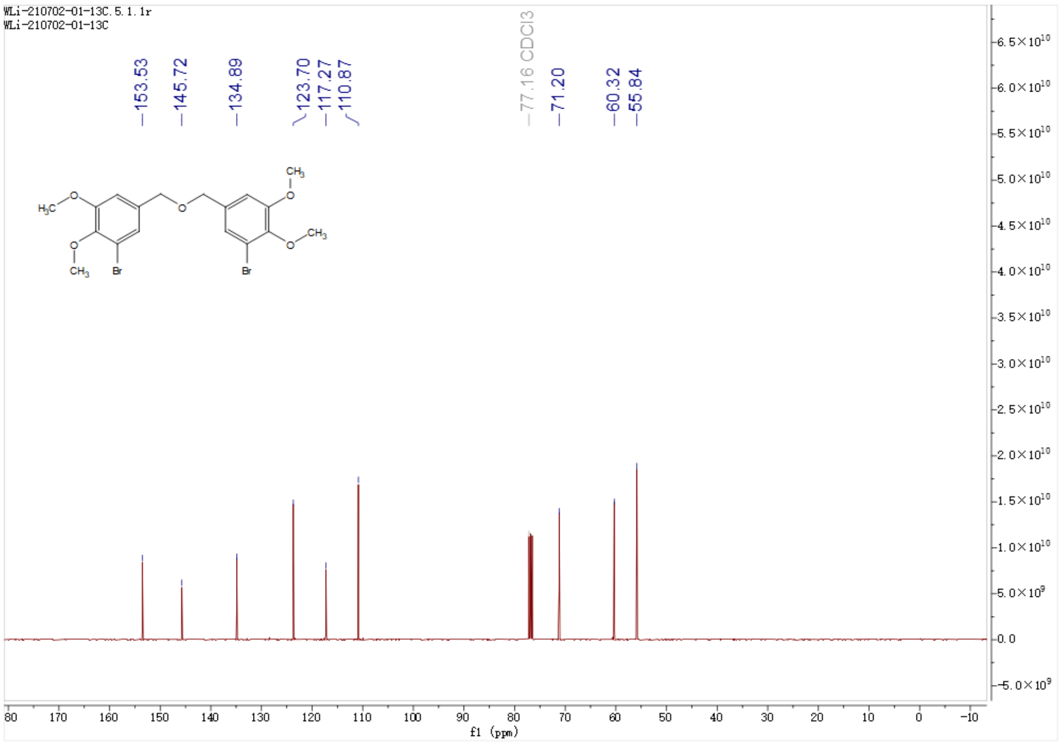

Figure S10. ^{13}C NMR spectrum of compound **3b-4** in Chloroform-*d* (101 MHz).

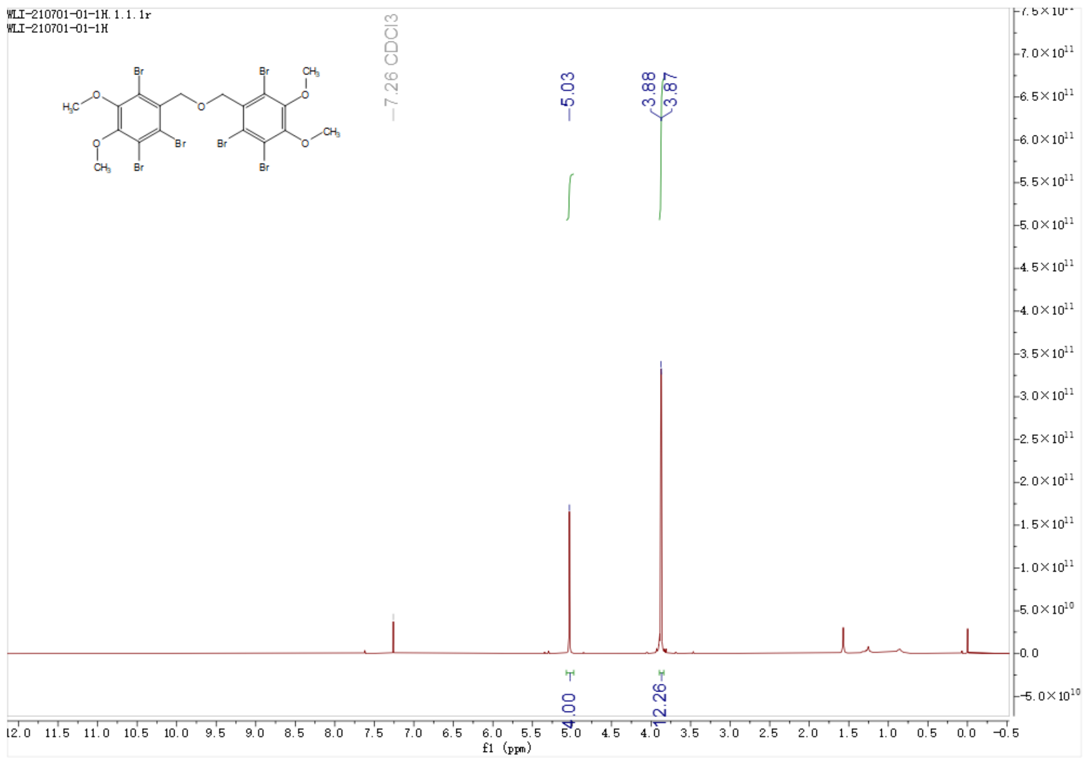

Figure S11. ^{1}H NMR spectrum of compound **3b-5** in Chloroform-*d* (400 MHz).

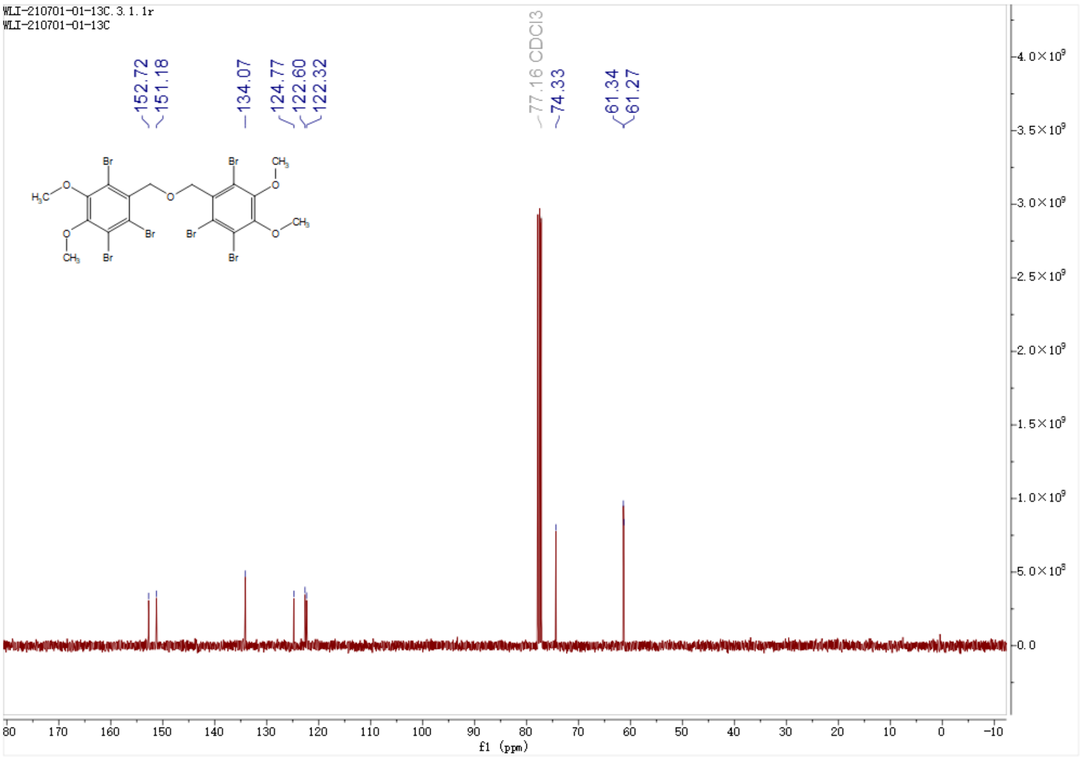

Figure S12. ^{13}C NMR spectrum of compound **3b-5** in Chloroform-*d* (101 MHz).

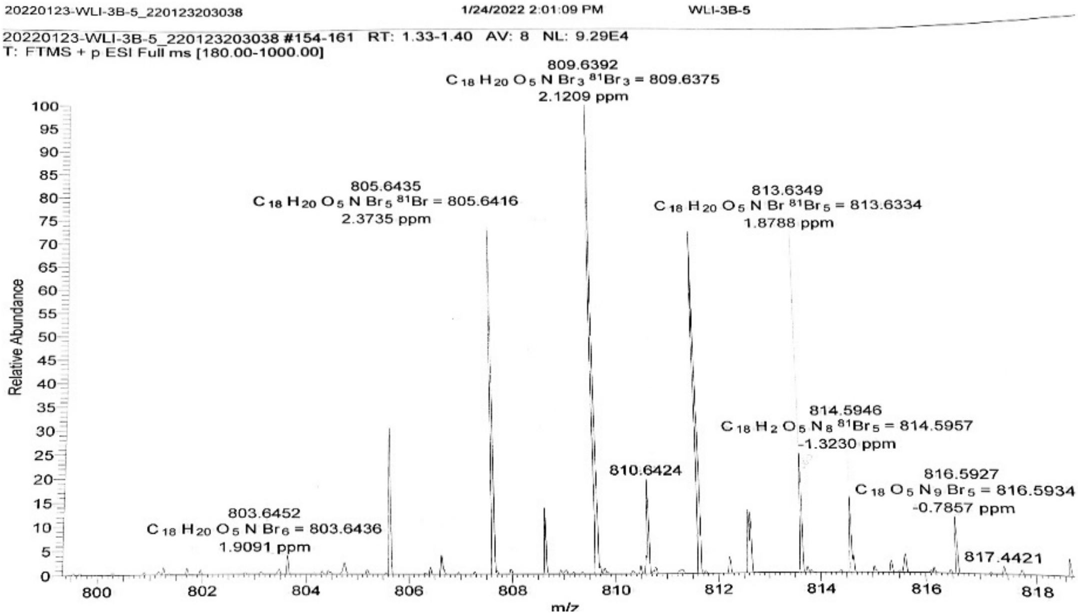

Figure S13. HRMS Spectra of 3b-5.

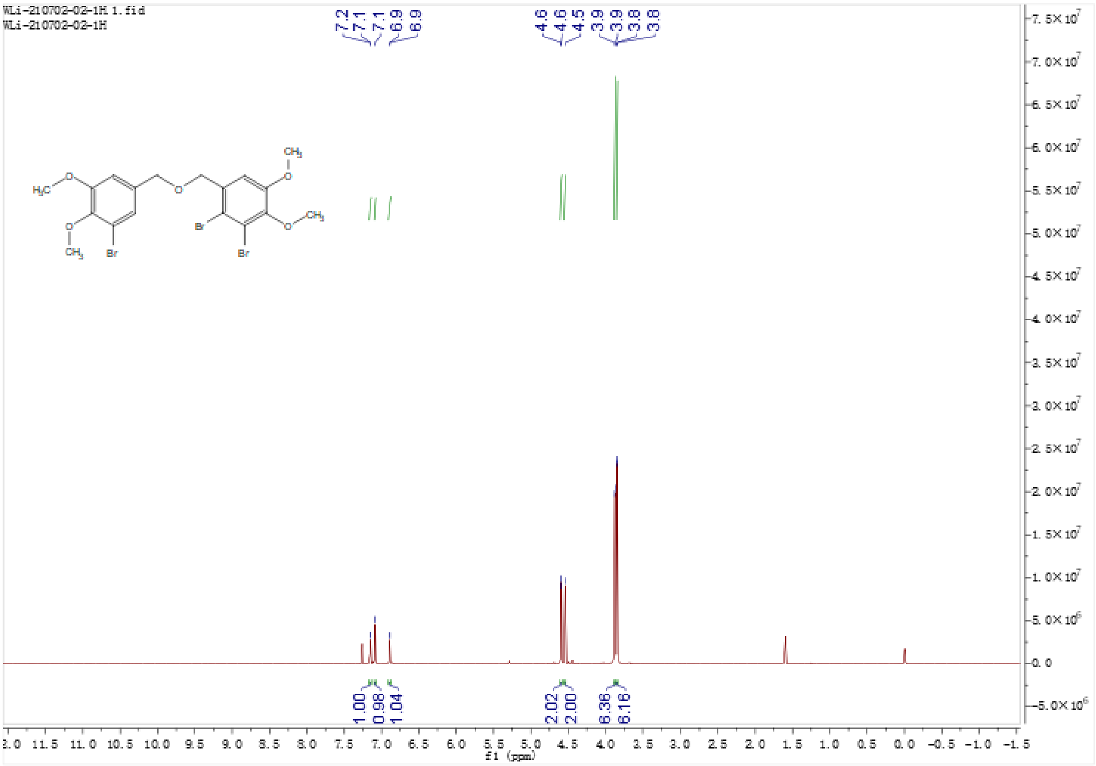

Figure S14. ^{1}H NMR spectrum of compound **3b-6** in Chloroform-*d* (400 MHz).

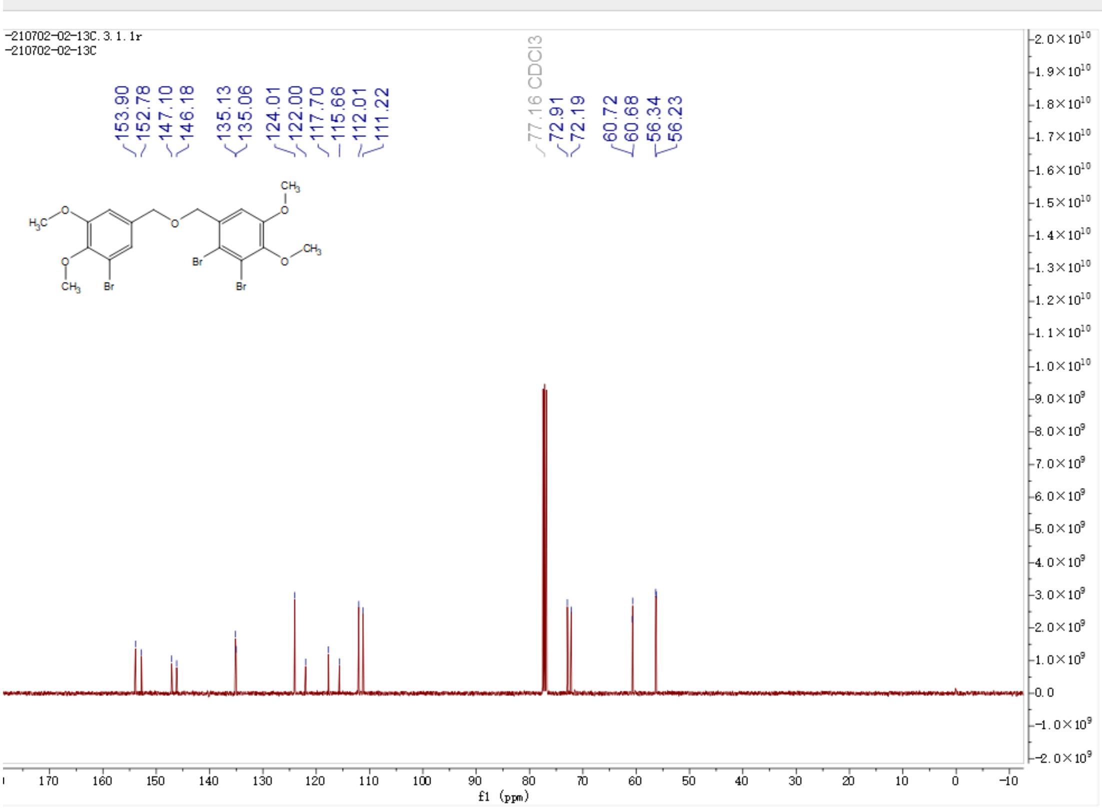

Figure S15. ^{13}C NMR spectrum of compound **3b-6** in Chloroform-*d* (101 MHz).

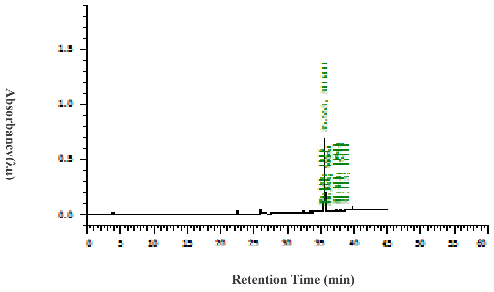

No.	RT	Area	Concentration	BC
1	35.020	1186	0.037	BB
2	35.233	1344	0.042	BB
3	35.553	3086778	95.854	BV
4	35.920	5551	0.172	TBB
5	36.233	12258	0.381	VB
6	37.007	17228	0.535	BV
7	37.327	49522	1.538	VB
8	37.673	14119	0.438	BV
9	38.040	15276	0.474	VV
10	38.873	17017	0.528	VB
		3220279	100.000	

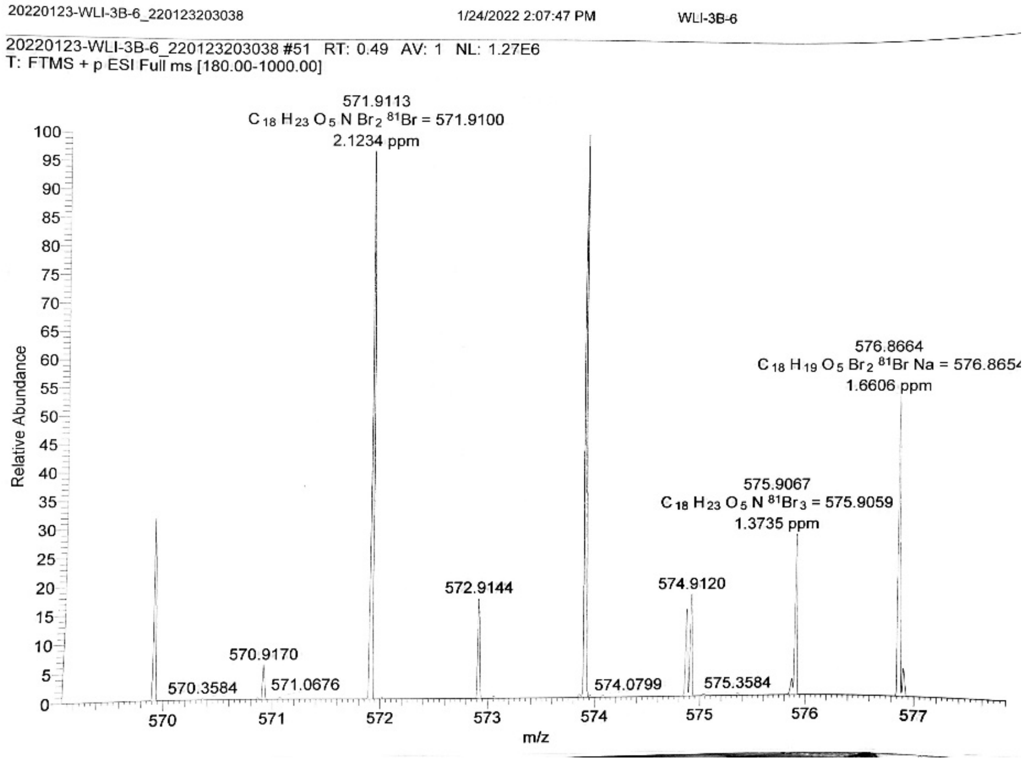

Figure S16. HRMS and HPLC Spectra of **3b-6.**

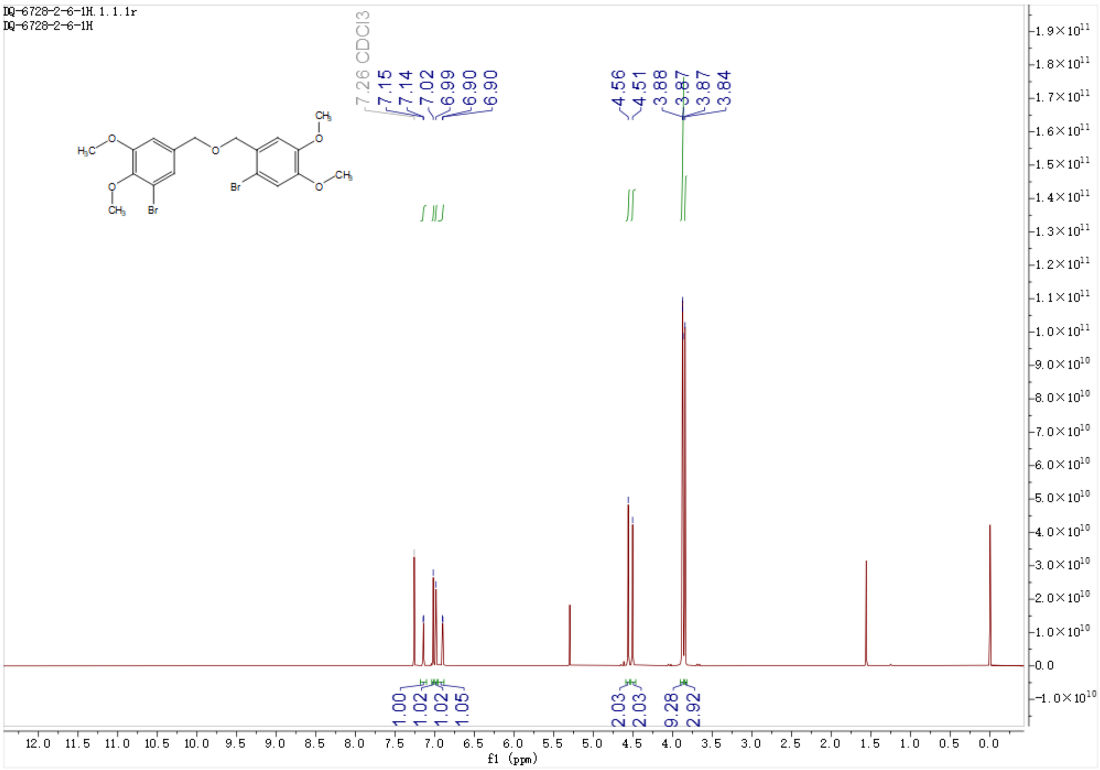

Figure S17. ^{1}H NMR spectrum of compound **3b-7** in Chloroform-*d* (400 MHz).

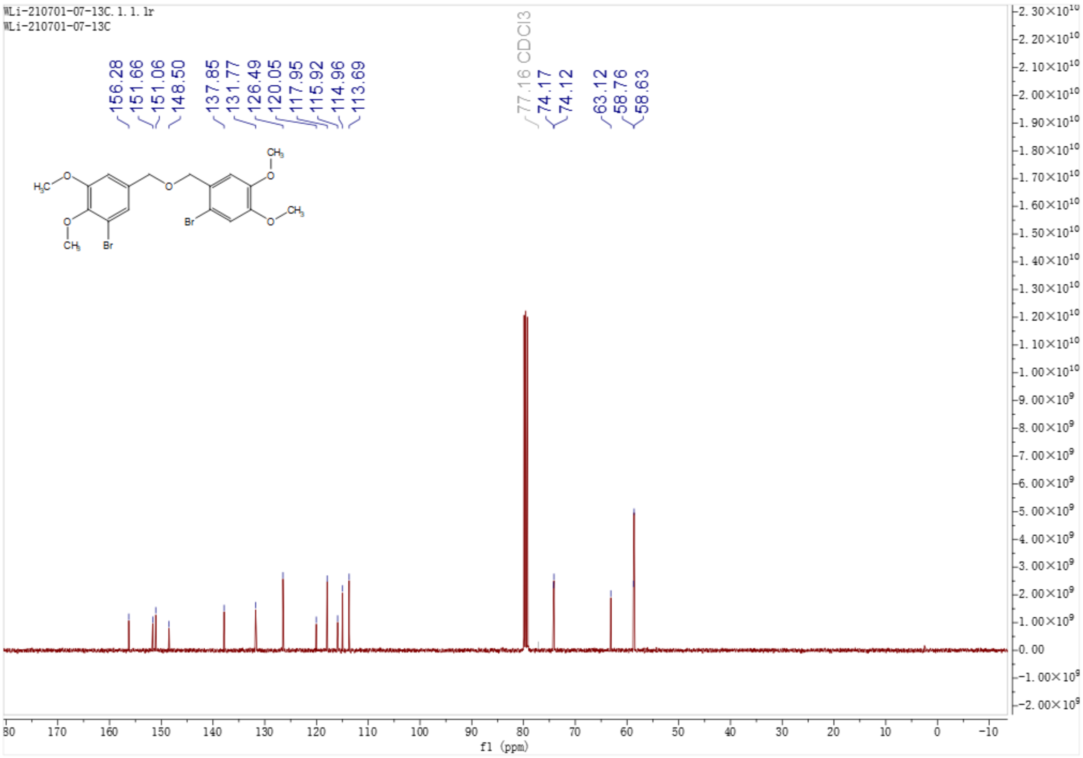

Figure S18. ^{13}C NMR spectrum of compound **3b-7** in Chloroform-*d* (101 MHz).

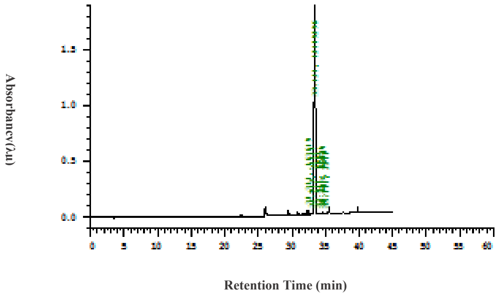

No.	RT	Area	Concentration	BC
1	32.380	163649	1.249	BB
2	32.973	52727	0.402	BV
3	33.420	12789296	97.577	VV
4	33.920	6662	0.051	TBB
5	34.387	21238	0.162	VV
6	34.613	63740	0.486	VV
7	34.860	7316	0.056	VV
8	35.047	2287	0.017	VB
		13106915	100.000	

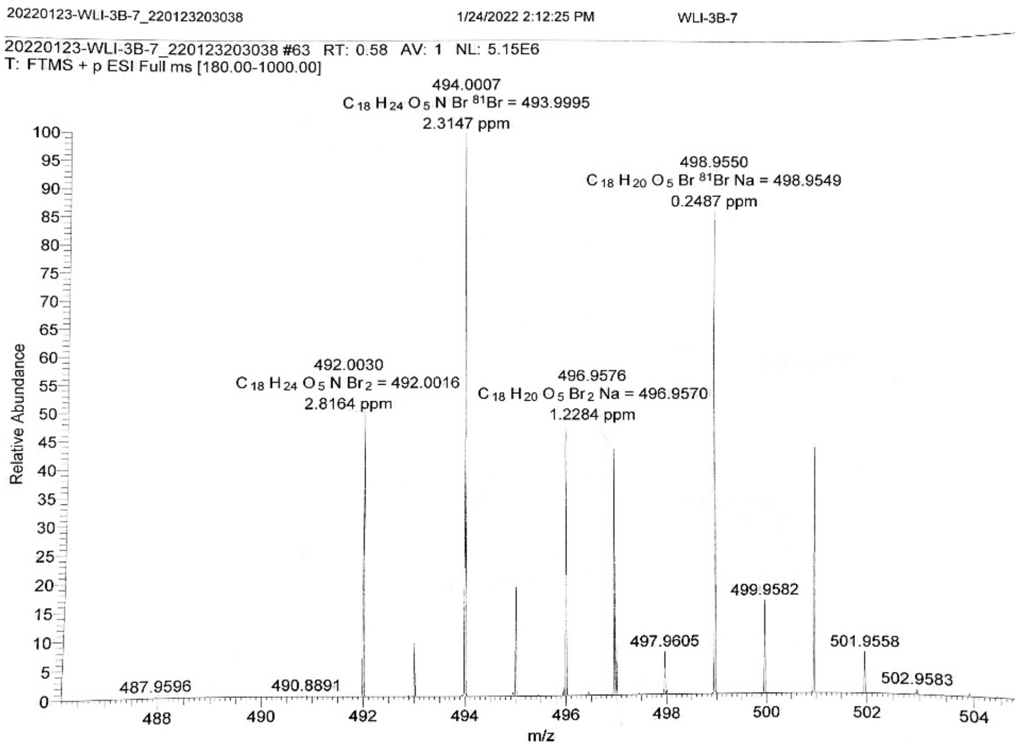

Figure S19. HRMS and HPLC Spectra of **3b-7.**

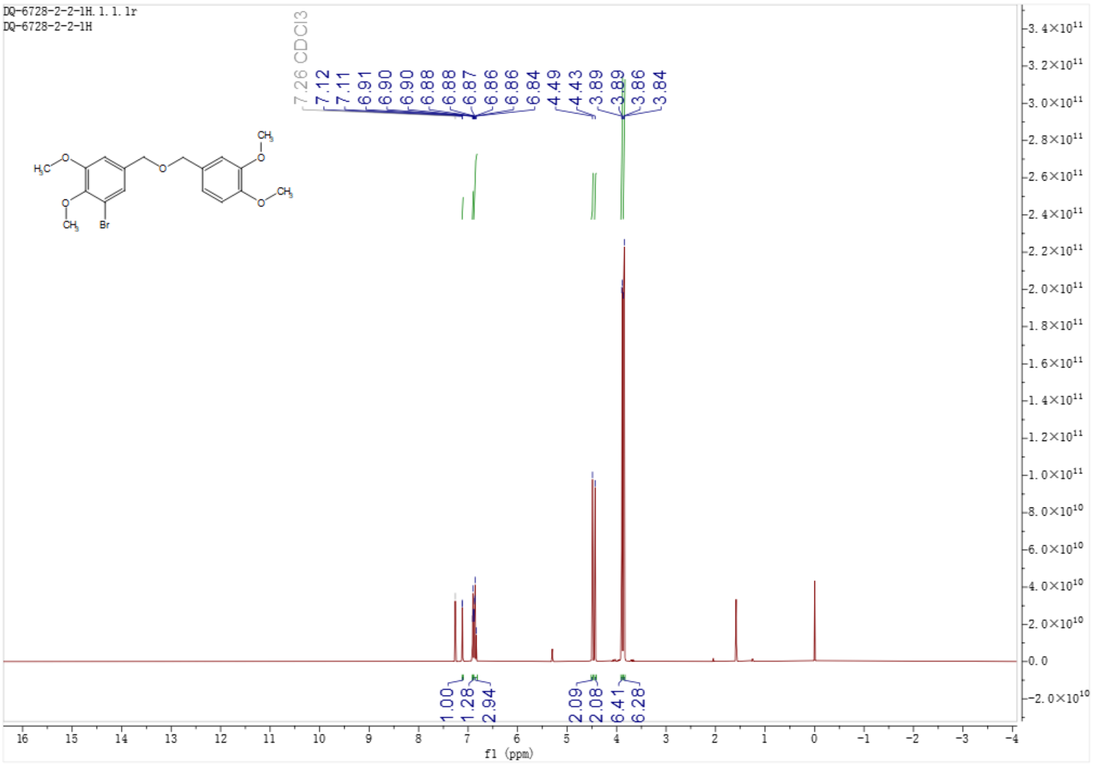

Figure S20. ^{1}H NMR spectrum of compound **3b-8** in Chloroform-*d* (400 MHz).

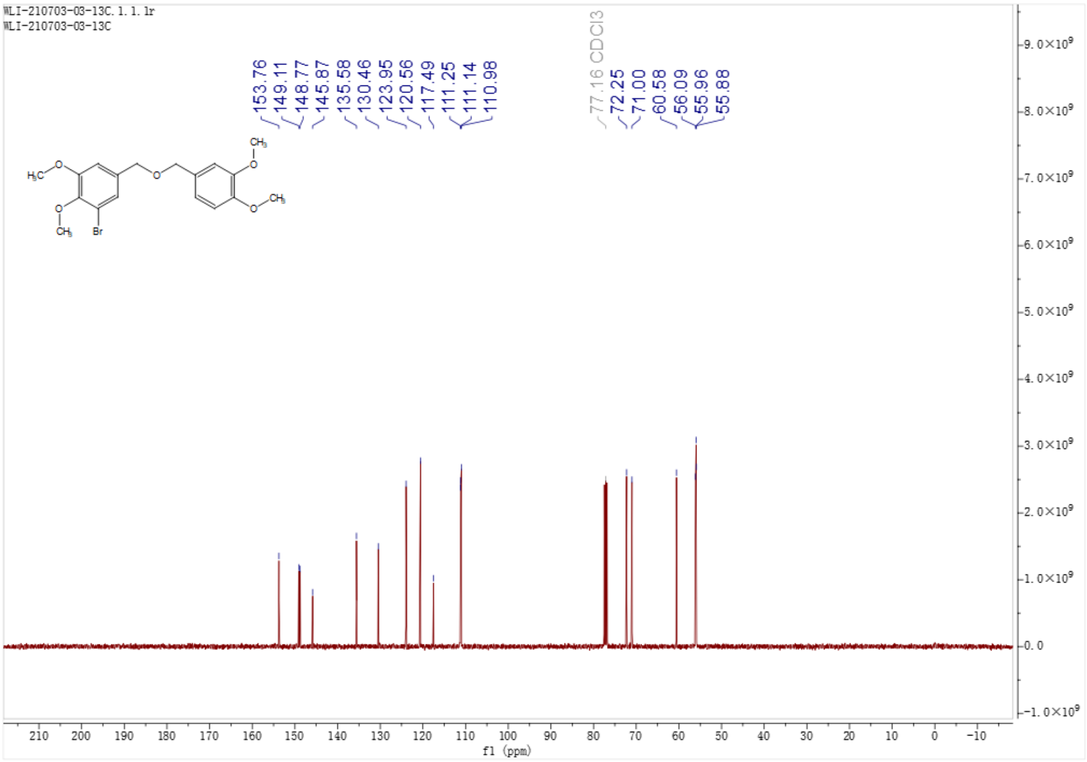

Figure S21. ^{13}C NMR spectrum of compound **3b-8** in Chloroform-*d* (101 MHz).

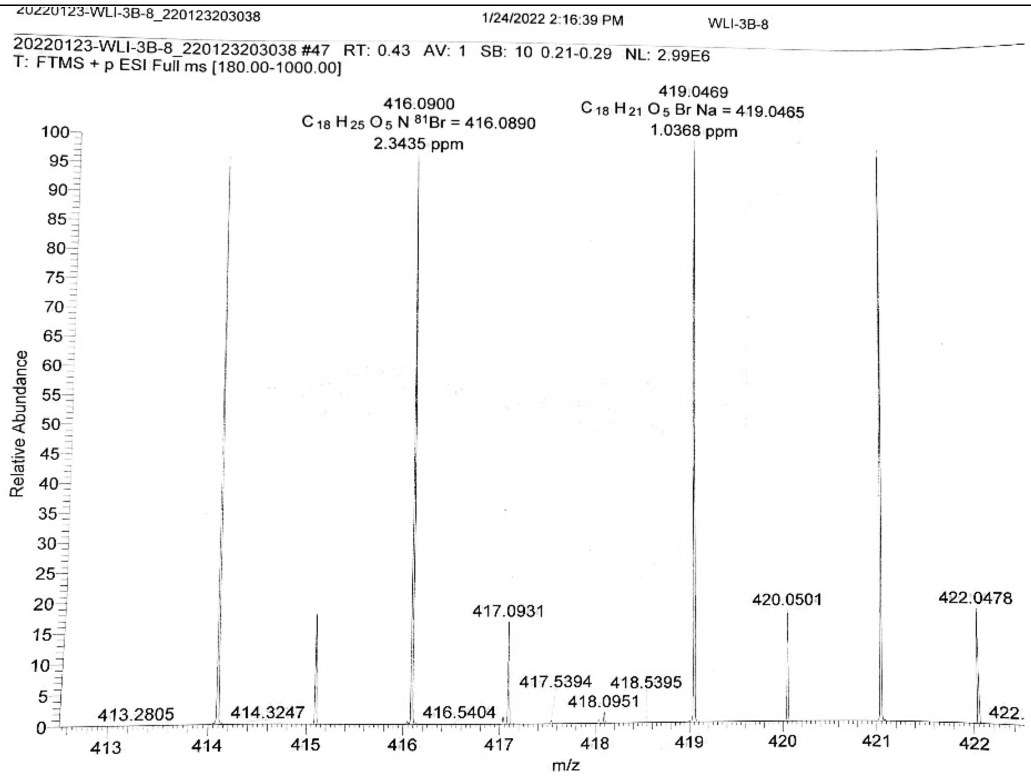

Figure S22. HRMS Spectra of 3b-8.

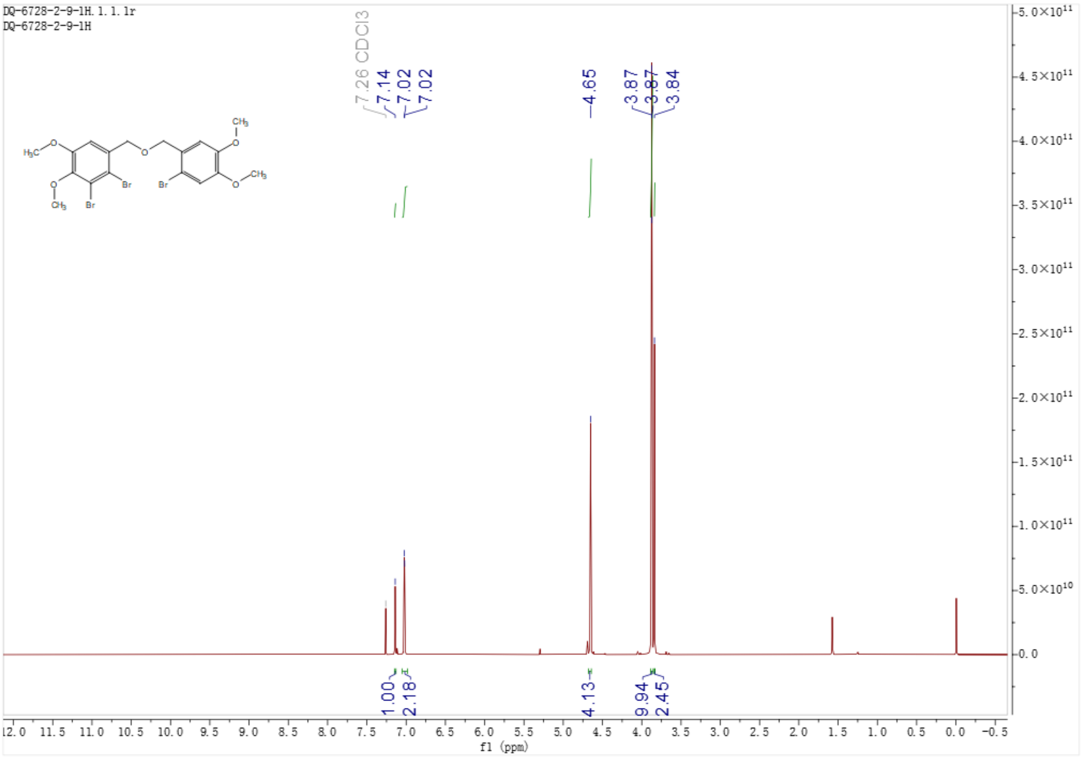

Figure S23. ^{1}H NMR spectrum of compound **3b-9** in Chloroform-*d* (400 MHz).

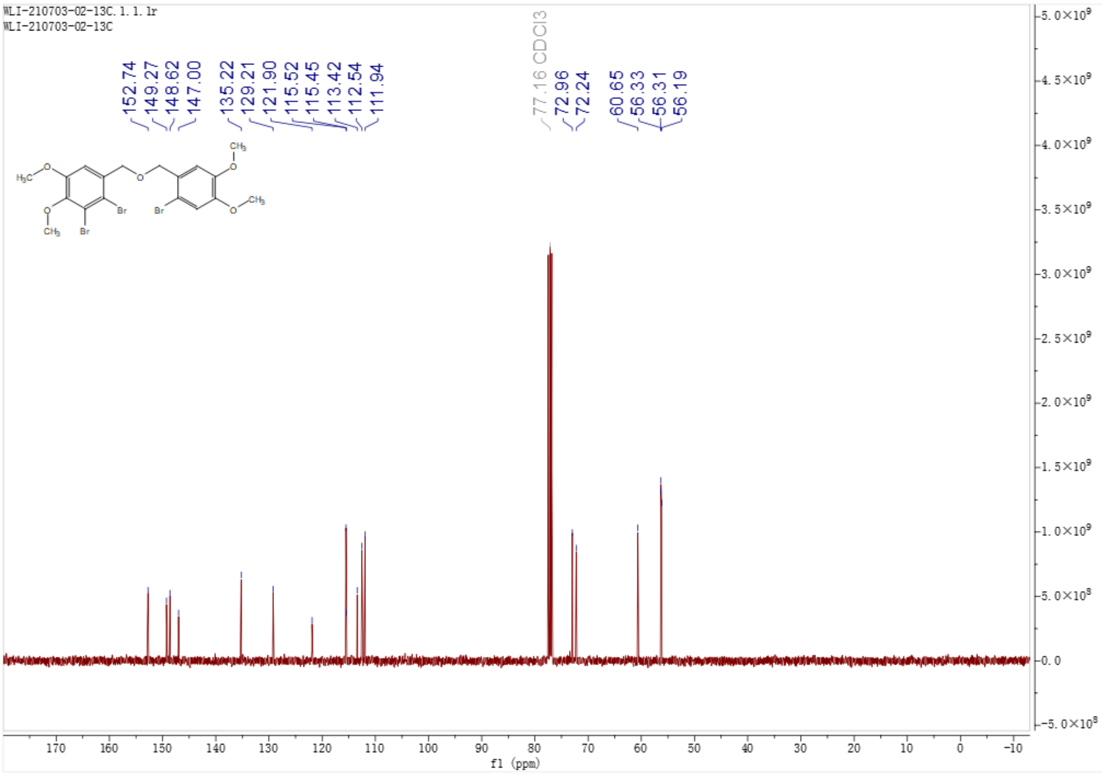

Figure S24. ^{13}C NMR spectrum of compound **3b-9** in Chloroform-*d* (101 MHz).

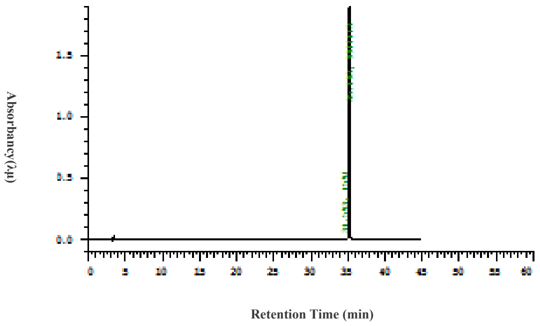

No.	RT	Area	Concentration	BC
1	34.593	7592	0.077	BV
2	35.193	9900743	99.923	VB
		9908335	100.000	

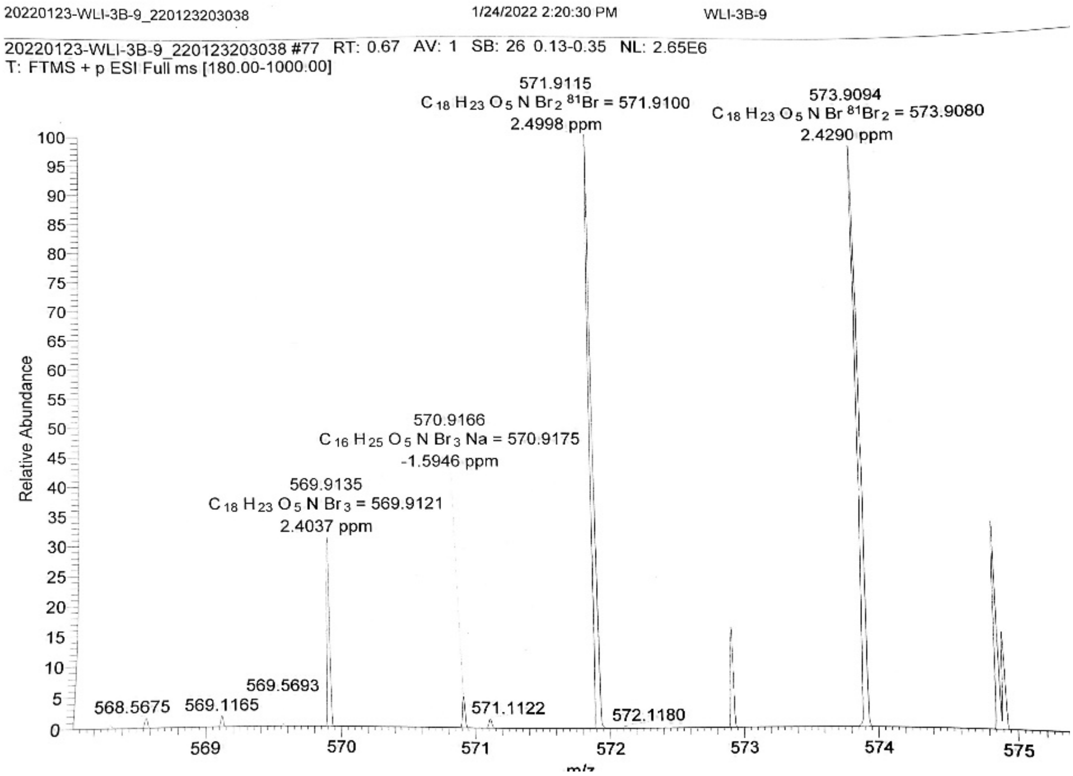

Figure S25. HRMS and HPLC Spectra of **3b-9.**

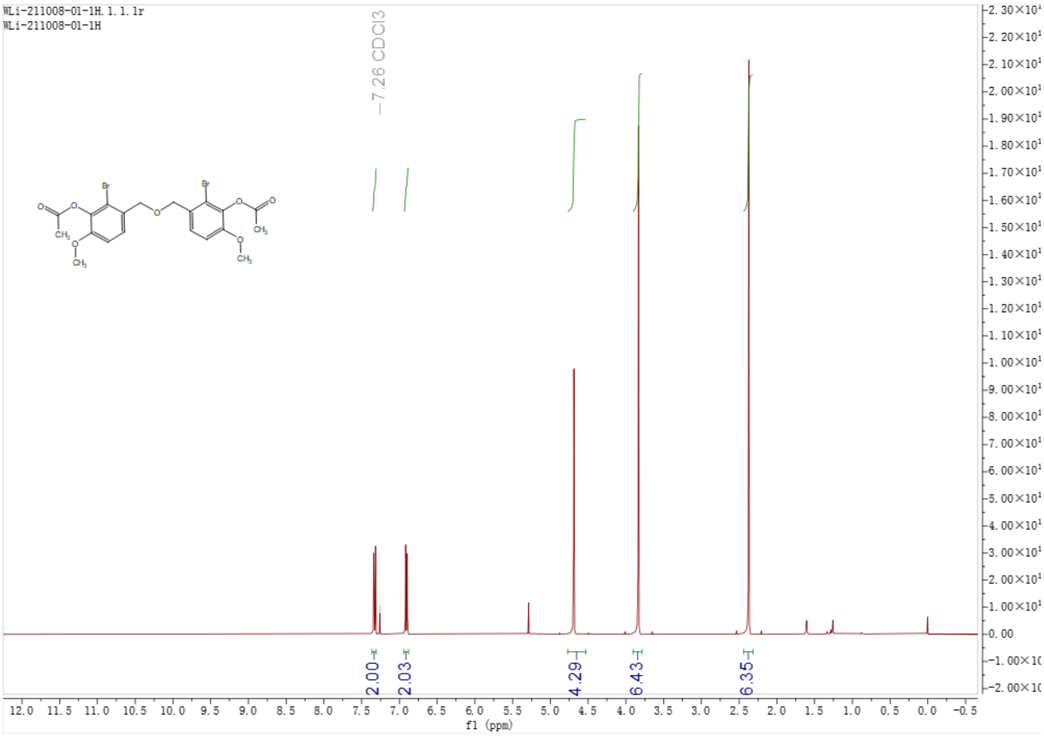

Figure S26. ^{1}H NMR spectrum of compound **4b-1** in Chloroform-*d* (400 MHz).

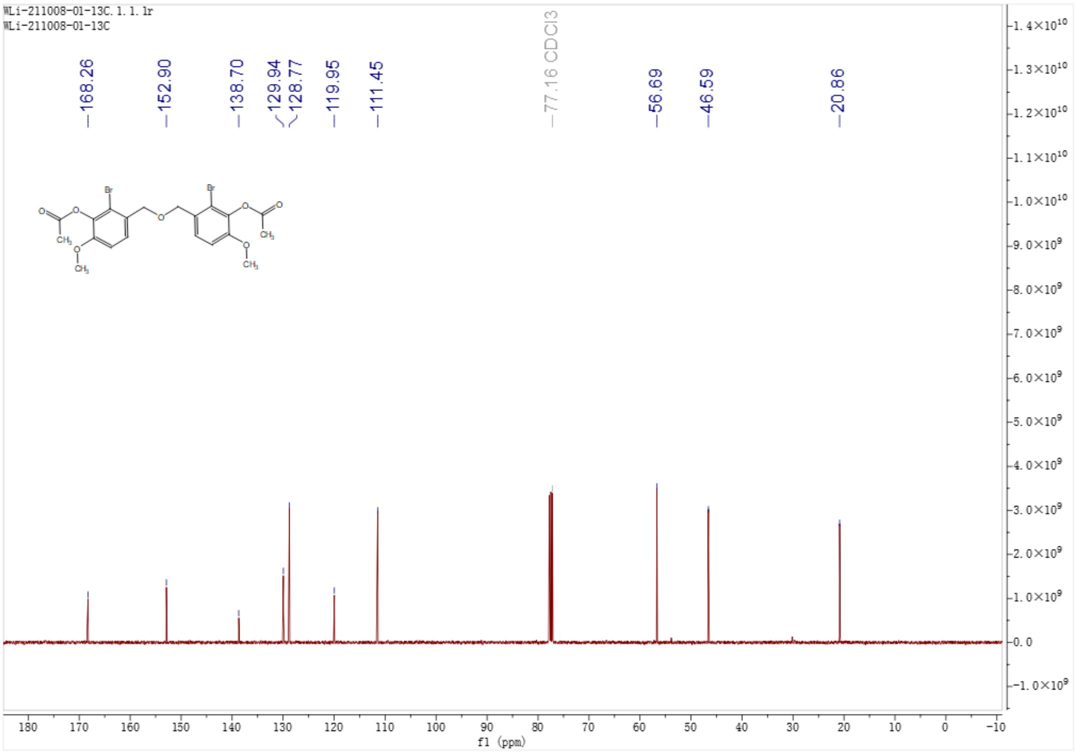

Figure S27. ^{13}C NMR spectrum of compound **4b-1** in Chloroform-*d* (101 MHz).

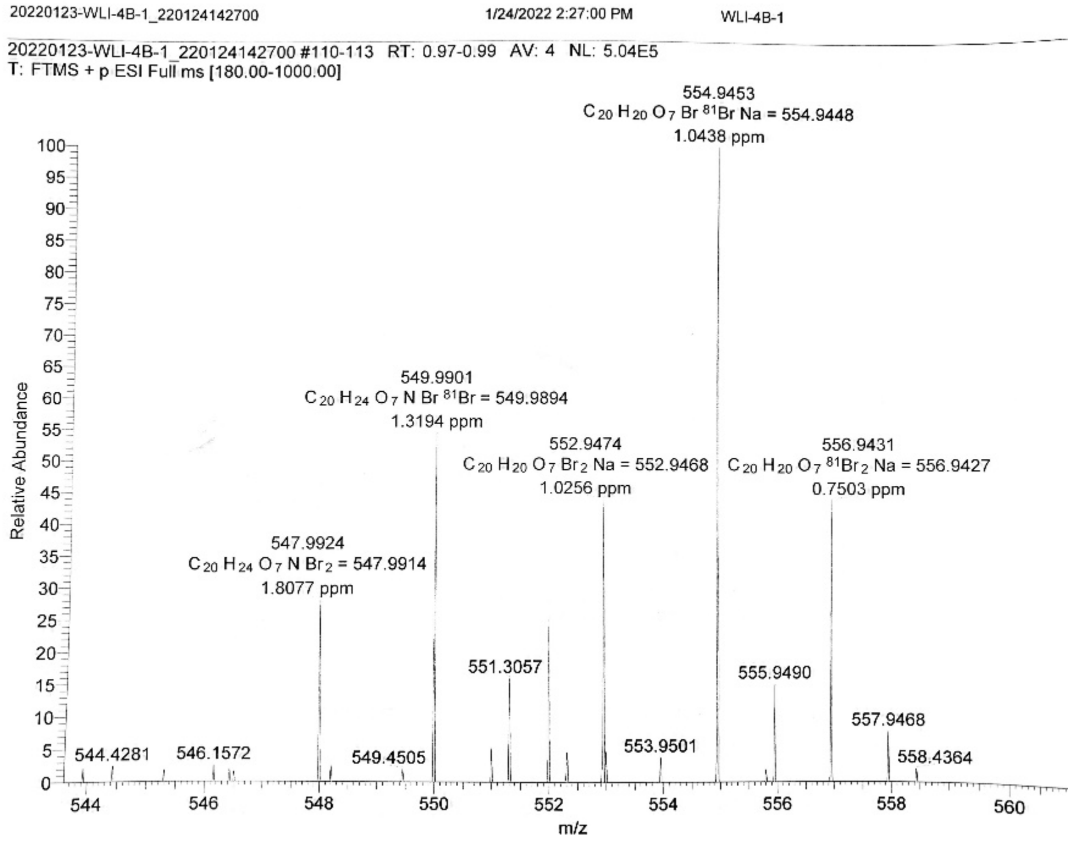

Figure S28. HRMS Spectra of 4b-1.

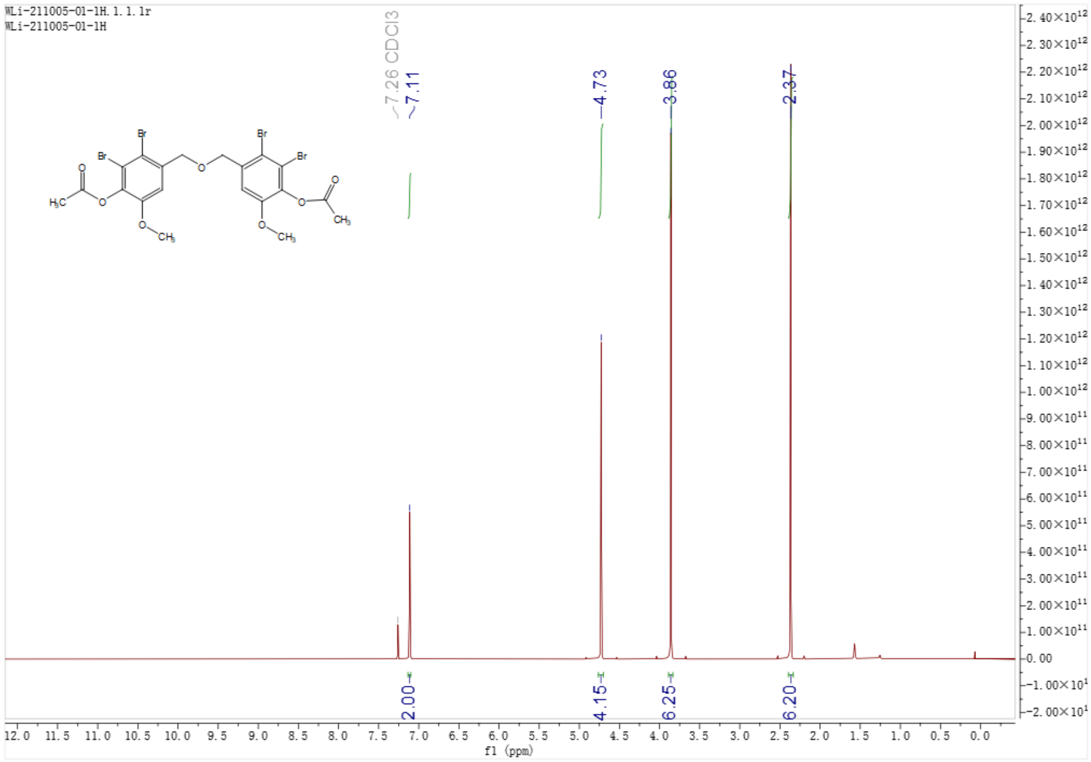

Figure S29. ^{1}H NMR spectrum of compound **4b-2** in Chloroform-*d* (400 MHz).

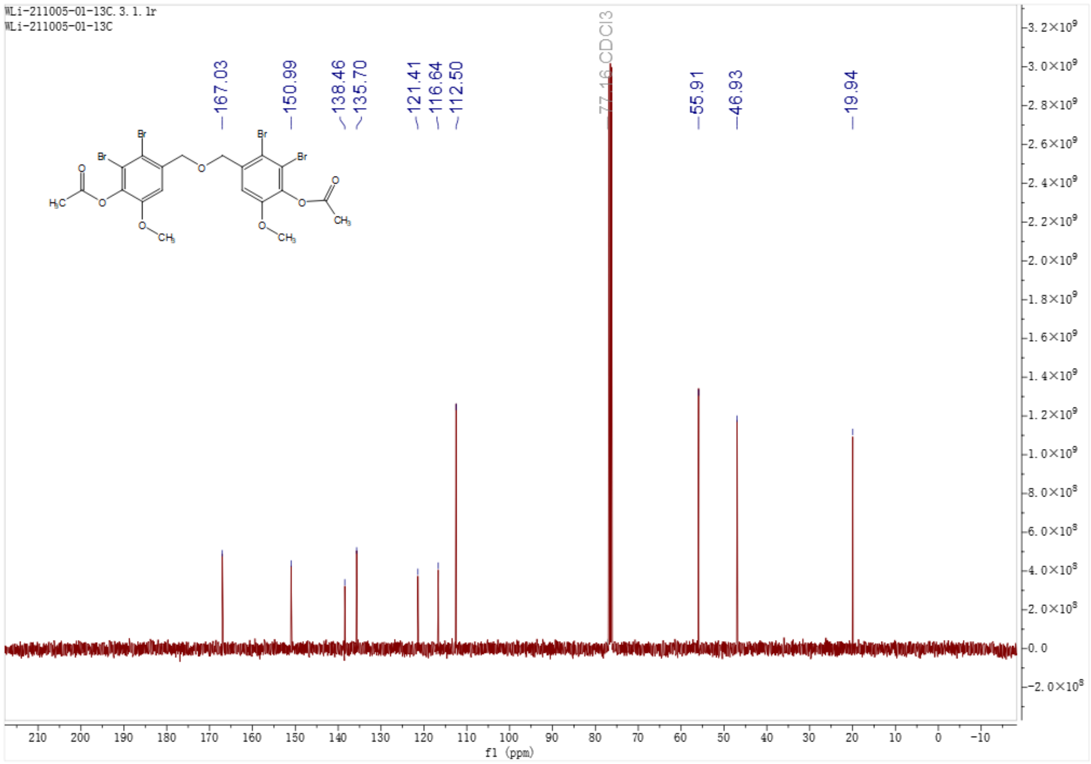

Figure S30. ^{13}C NMR spectrum of compound **4b-2** in Chloroform-*d* (101 MHz).

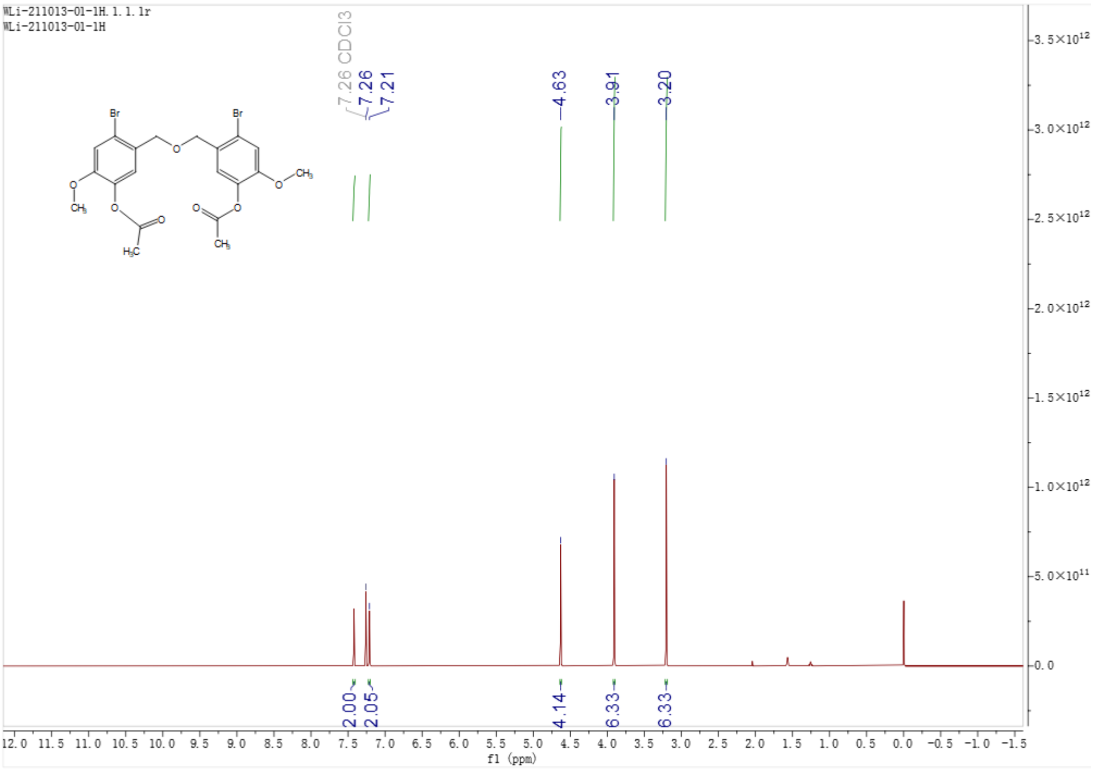

Figure S31. ^{1}H NMR spectrum of compound **4b-3** in Chloroform-*d* (400 MHz).

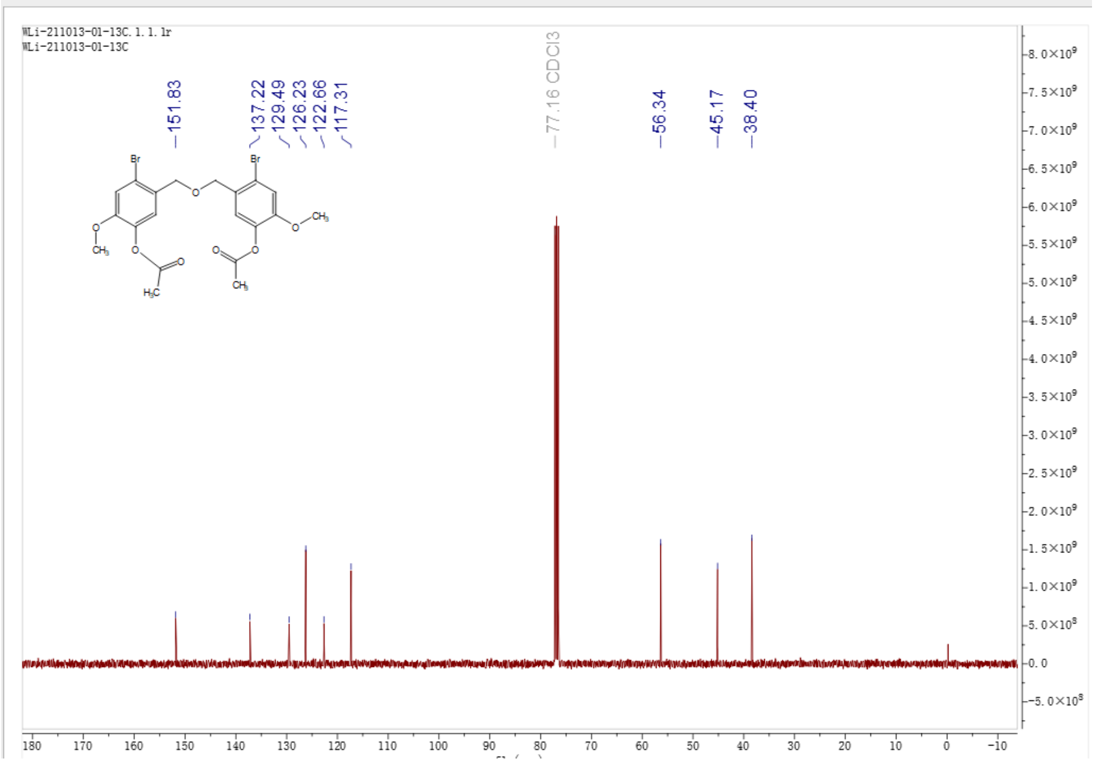

Figure S32. ^{13}C NMR spectrum of compound **4b-3** in Chloroform-*d* (101 MHz).

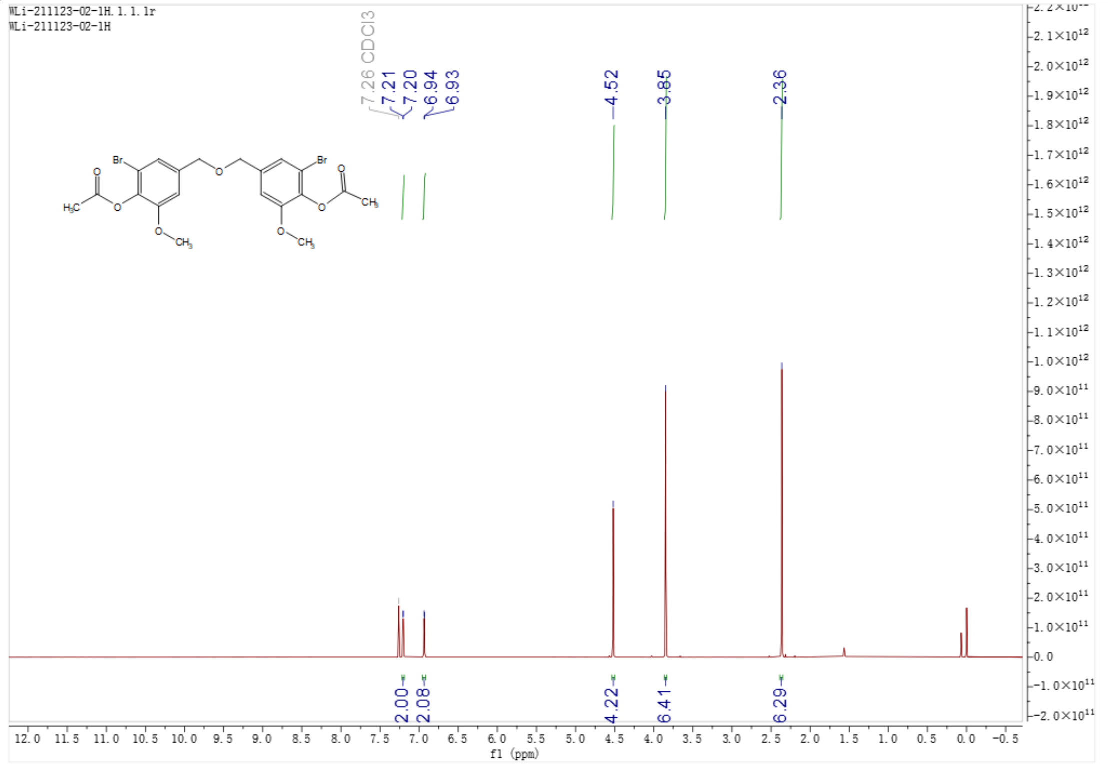

Figure S33. ^{1}H NMR spectrum of compound **4b-4** in Chloroform-*d* (400 MHz).

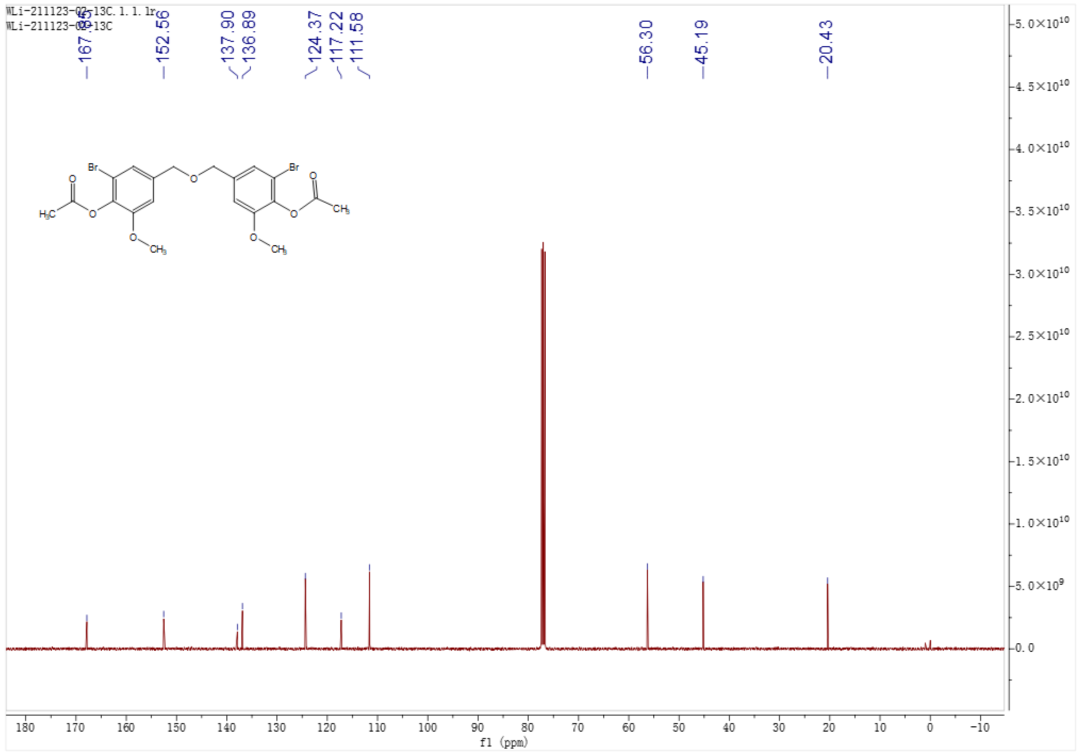

Figure S34. ^{13}C NMR spectrum of compound **4b-4** in Chloroform-*d* (101 MHz).

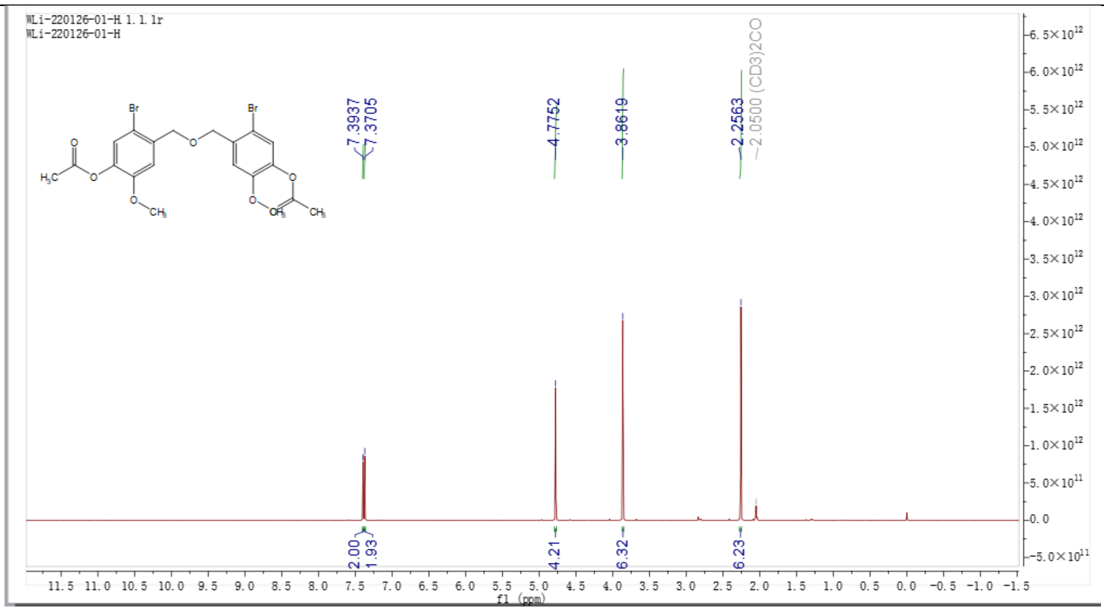

Figure S35. ^{1}H NMR spectrum of compound **4b-5** in Chloroform-*d* (400 MHz).

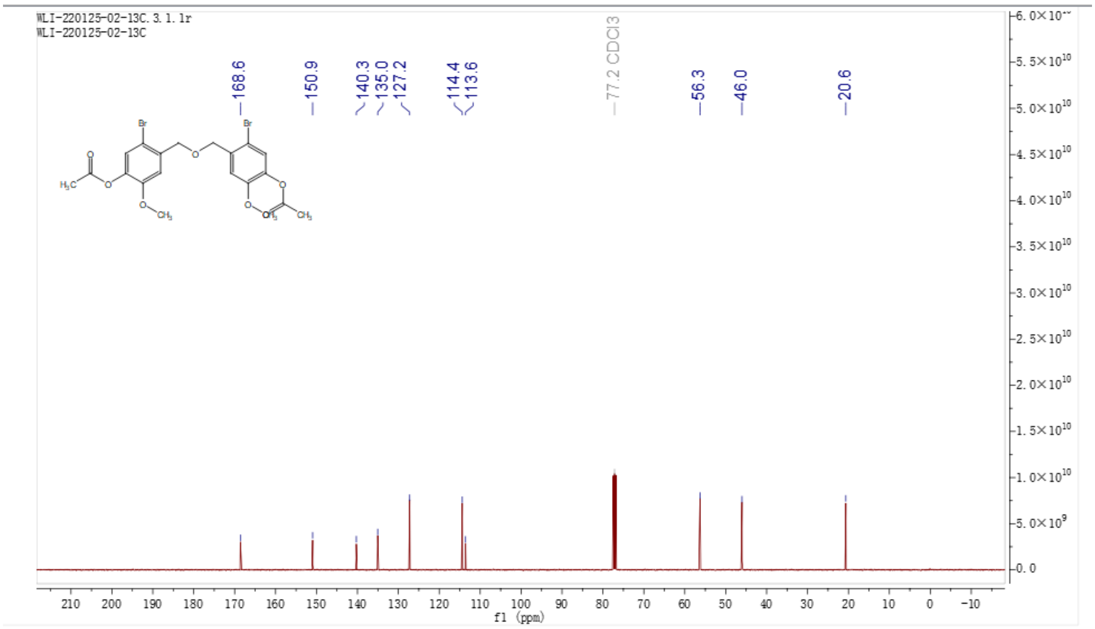

Figure S36. ^{13}C NMR spectrum of compound **4b-5** in Chloroform-*d* (101 MHz).

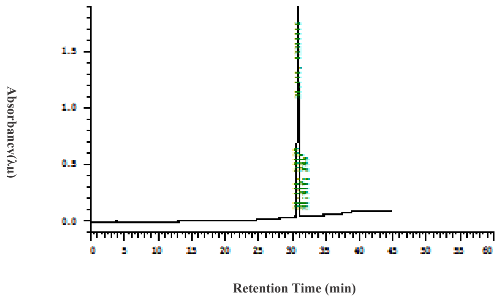

No.	RT	Area	Concentration	BC
1	30. 427	59839	0. 447	BV
2	30. 847	13318176	99. 509	VV
3	31. 313	5377	0. 040	TBB
4	31. 687	232	0. 002	TBB
5	31. 980	299	0. 002	BB
		13383923	100. 000	

Figure S37. HRMS Spectra of 4b-5.

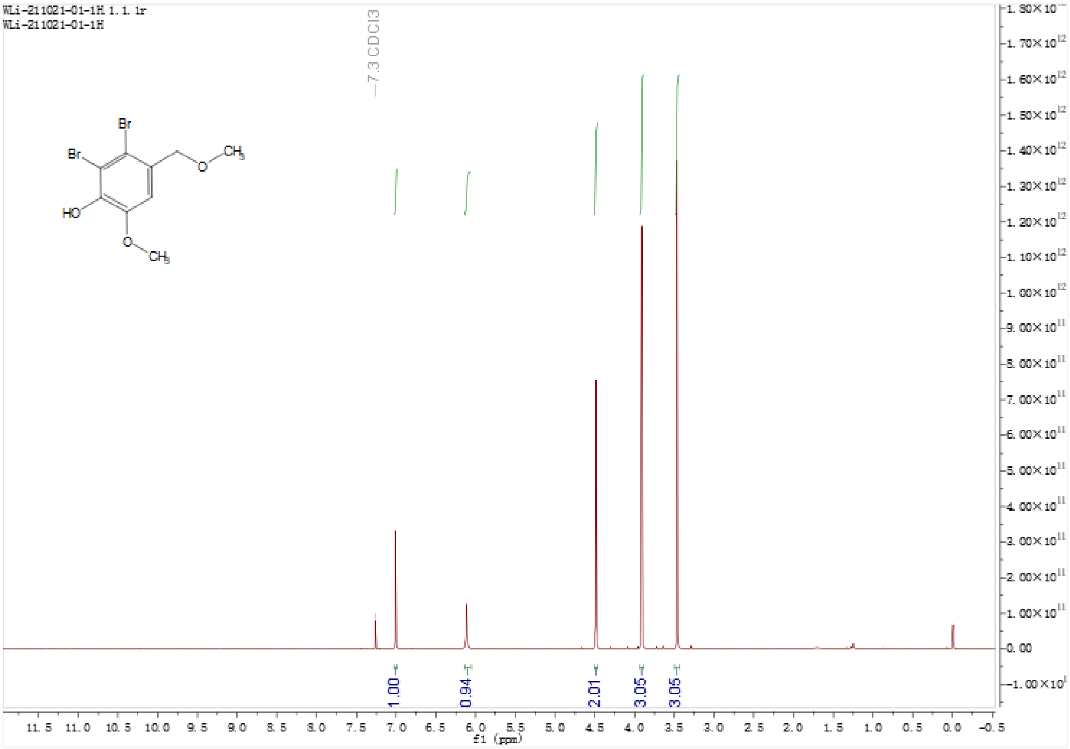

Figure S38. ^{1}H NMR spectrum of compound **4b-6** in Chloroform-*d* (400 MHz).

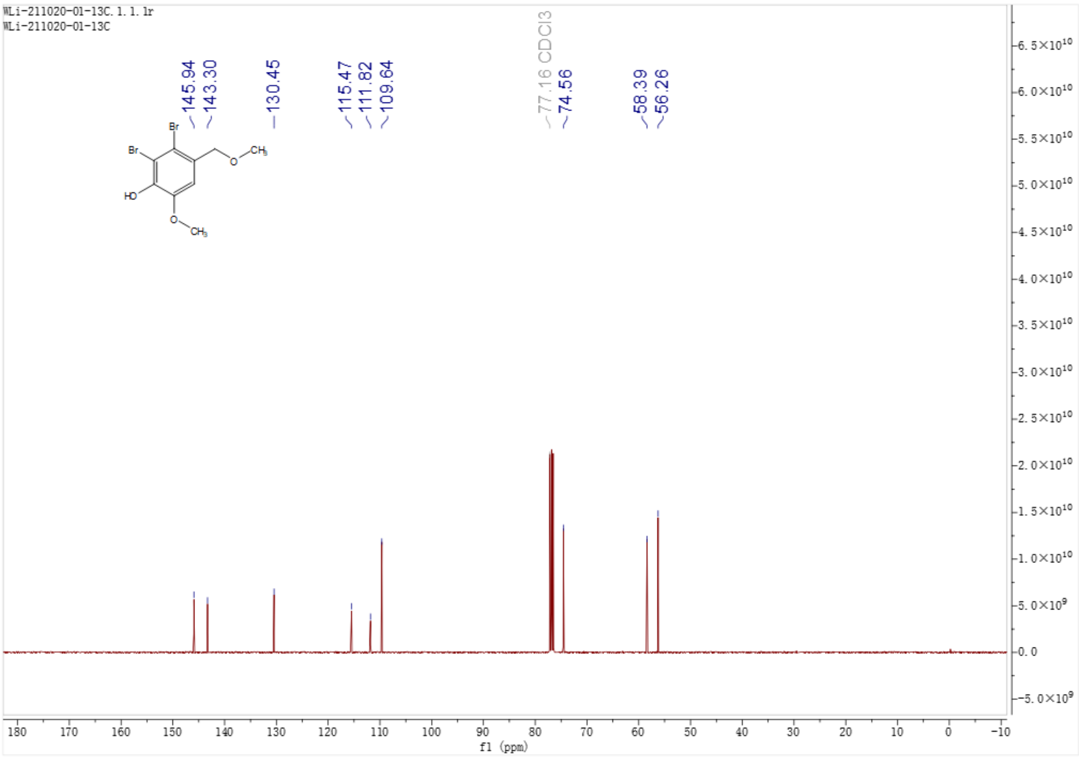

Figure S39. ^{13}C NMR spectrum of compound **4b-6** in Chloroform-*d* (101 MHz).

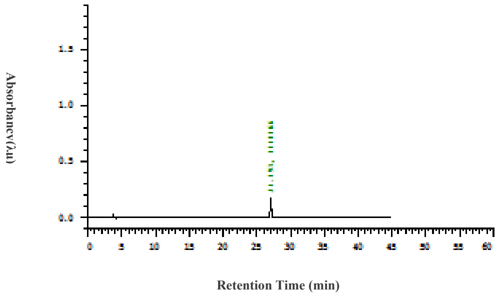

No.	RT	Area	Concentration	BC
1	27.093	1048066	100.000	BB
		1048066	100.000	

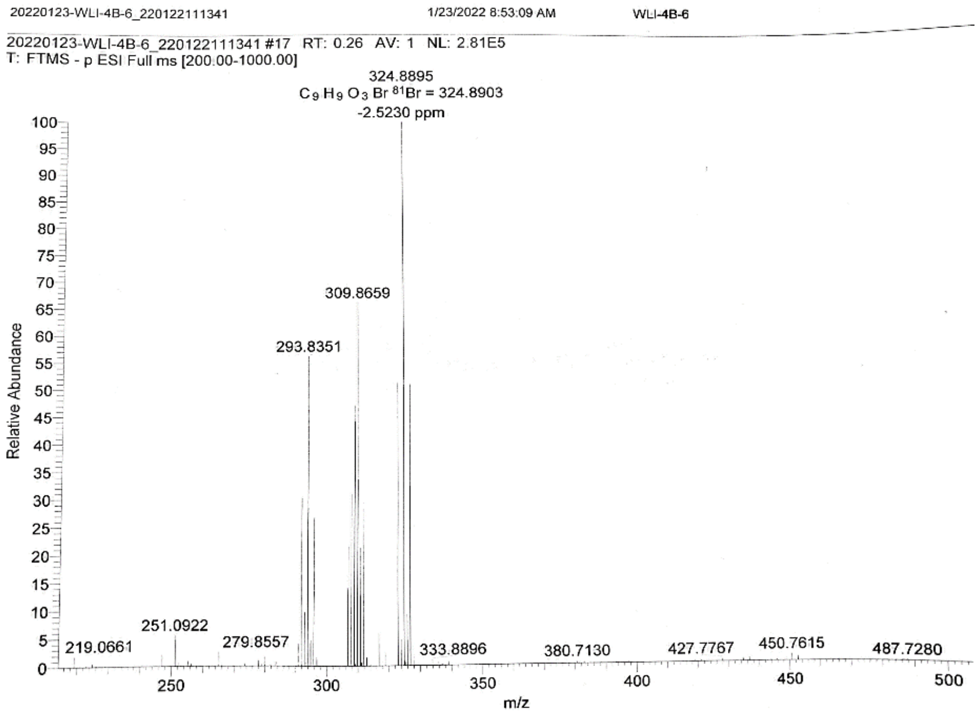

Figure S40. HRMS and HPLC Spectra of **4b-6.**
